# Upcycling Industrial Lignin Into Cationic Hydrogels With Potential for Sustainable Plant Protection

**DOI:** 10.1002/cssc.71125

**Published:** 2026-10-15

**Authors:** Darindra Sandya Thanos, Zisan Caliskan Acik, Emma Pauline Becker, Max‐Emanuel Zirngibl, Tatyana L. Povolotsky, Marcel Wiermer, Mitja Remus‐Emsermann, Sanjam Chandna

**Affiliations:** ^1^ Institute of Chemistry and Biochemistry Freie Universität Berlin Berlin Germany; ^2^ Institute of Biology Freie Universität Berlin Berlin Germany

**Keywords:** antimicrobial materials, cationic lignin, chitosan–genipin hydrogel, plant protection, *Pseudomonas syringae*

## Abstract

Bio‐based antimicrobial materials are increasingly explored as sustainable alternatives for plant pathogen control. Here, we report a circular economy approach in which industrial kraft lignin was upcycled into cationic lignin (CL) and physically embedded within chitosan–genipin (CH–GPN) hydrogels to form CH–GPN–CL. These bioactive materials were evaluated for the in vitro inhibition of the plant pathogen *Pseudomonas syringae* pv. *tomato* DC3000. To enhance antimicrobial activity, kraft lignin was cationized with glycidyl trimethylammonium chloride (GTMAC) using different lignin/GTMAC mass ratios, producing CL samples with varying degrees of substitution (DS). Leaf infiltration‐based phytotoxicity assays of CL using *Nicotiana benthamiana* showed no concentration‐ or DS‐dependent phytotoxicity under buffered conditions, suggesting compatibility with infiltrated leaf tissue. Bacterial inhibition assays showed that GTMAC cationization strongly improved lignin antimicrobial activity. Based on strong activity and a lower lignin/GTMAC mass ratio, a representative CL sample was selected for incorporation into the hydrogel framework. The resulting CH–GPN–CL hydrogels retained gel‐like behavior and porous morphology and showed enhanced in vitro antimicrobial activity compared to the control hydrogel without embedded CL. These findings highlight the potential of CH–GPN–CL hydrogels as sustainable bioactive materials for further plant protection evaluation.

## Graphical abstract

Industrial lignin is upcycled into cationic lignin (CL) and embedded within chitosan–genipin (CH–GPN) hydrogels to form bioactive hydrogel networks with potential for antimicrobial plant protection. The CL‐embedded CH–GPN hydrogels retain porous, gel‐like properties and show enhanced in vitro inhibition of the plant pathogen *Pseudomonas syringae*.

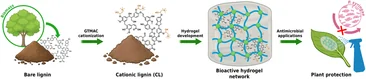

## 1 Introduction

The increasing demand for sustainable agricultural practices has created a need for safer and more efficient alternatives to conventional chemical pesticides. Many conventional pesticides are associated with environmental concerns, including persistence in soil and water [1], nontarget toxicity [2], and harmful effects on public health [3]. Their repeated use has also led to the development of resistant bacterial strains [4, 5], reducing the efficacy of the pesticides in long‐term use. In foliar applications, spray‐based pesticides can suffer poor retention on leaf surfaces, especially under rain, which increases the risk of leaching into the soil and aquatic environments [6]. These challenges have motivated interest in developing bio‐based antimicrobial materials and formulation strategies that can improve local antimicrobial activity and remain compatible with plant tissue.

In this context, lignin, an abundant and renewable byproduct of the pulp and paper industry, represents an attractive starting material for a bio‐based antimicrobial material. Despite its availability, lignin remains largely underutilized, with most of it being incinerated for energy rather than converted into higher‐value functional materials [7, 8]. One promising approach for valorizing lignin is through the introduction of cationic groups onto its backbone, for example, through cationization with quaternary ammonium groups [9, 10, 11]. Compounds containing quaternary ammonium groups have exhibited antimicrobial activity through electrostatic interactions with negatively charged microbial membranes that lead to membrane disruption and cell death [12, 13]. In addition, previous studies have also shown that lignin cationized with quaternary ammonium groups can act against a range of microorganisms, demonstrating the effectiveness of this cationization approach [14, 15]. However, these investigations have mainly focused on pathogens relevant to mammalian systems, while the potential of cationic lignin (CL) in plant protection remains relatively unexplored.

For plant‐related applications, antimicrobial activity must be balanced with plant compatibility. Materials should not induce phytotoxic effects and should remain effective against relevant plant pathogens, such as *Pseudomonas syringae*, a widespread bacterial plant pathogen [16, 17]. It has several pathovars that infect many agriculturally important crops worldwide, with disease outbreaks reported across annual crops and fruit‐tree systems [18, 19]. Since cationic antimicrobial materials, such as CL, may also interact with plant tissues, both the antimicrobial performance and plant compatibility of CL need to be evaluated.

In addition to the antimicrobial material itself, the formulation strategy is also important for practical applications. Hydrogel‐based systems offer a versatile platform for incorporating and delivering active compounds in a controlled manner. Their network structure can help retain embedded materials, regulate release, and maintain activity over time [20, 21]. Biopolymer‐based hydrogels are especially attractive in this context because they combine biocompatibility with tunable formulation properties. Chitosan–genipin (CH–GPN) hydrogels are one example of such a system [22, 23], where chitosan provides a cationic natural polymer backbone [24, 25], and genipin acts as a bio‐based cross‐linker to form a stable and biodegradable network [26, 27]. Chitosan is also known for its biocompatibility and its reported role in supporting plant protection [28, 29]. Together with the reported antimicrobial properties of genipin‐cross‐linked chitosan‐based hydrogels [25, 30], these features make CH‍–‍GPN hydrogels attractive candidates to investigate as biopolymer networks for carrying antimicrobial materials. In particular, hydrogels with tunable cross‐linking density and porosity can influence swelling behavior and the release of embedded compounds [31]. In future sprayable plant protection formulations, such matrix properties could help improve retention on leaf surfaces and reduce material loss to the surrounding environment [31, 32]. Therefore, embedding CL within a CH–GPN hydrogel network could combine the antimicrobial activity of CL with the retention and delivery properties of a bioactive hydrogel network.

Taken together, this makes CL‐embedded CH–GPN hydrogels a promising formulation concept, but the relevant literature remains fragmented. CL has been investigated as a bio‐based antimicrobial material, but previous studies have mainly focused on model microorganisms and human‐ or medically relevant pathogens rather than plant pathogenic bacteria [14, 15]. In parallel, lignin‐based materials have been explored in plant protection technologies, particularly as carriers or controlled‐release matrices for pesticides, fungicides, and herbicides [33, 34]. Chitosan‐based hydrogels have also been explored for agricultural applications, including pesticide delivery and antifungal activity against plant‐associated pathogens [35]. However, in most of these systems, lignin or other biopolymers mainly serve as formulation matrices for conventional agrochemical actives. In contrast, the direct use of GTMAC‐cationized lignin itself as the bioactive antibacterial component against plant pathogenic bacteria remains insufficiently explored. Therefore, this work focuses on evaluating the antibacterial activity and plant tissue compatibility of CL, using a CH–GPN network as a biopolymer hydrogel framework for its formulation and release.

In this work, we report the synthesis and evaluation of CL‍‐‍embedded CH–GPN hydrogels as bioactive hydrogel networks against the plant pathogenic *P. syringae* pv. *tomato* DC3000 strain. A library of CL samples with different degrees of substitution (DS) was prepared by reacting kraft lignin with glycidyl trimethylammonium chloride (GTMAC), allowing control over charge density and physicochemical properties. The materials were characterized and evaluated for their compatibility with plant tissue using phytotoxicity assays via leaf infiltration. For this purpose, *Nicotiana benthamiana* was used as a model plant due to its suitability for leaf infiltration experiments and its relevance in plant pathogen studies [36, 37, 38]. CL was then incorporated into CH–GPN hydrogels to explore their use as bioactive hydrogel matrices. Finally, the antimicrobial activity of both free CL and CL‐embedded hydrogels was evaluated against *P. syringae* pv. *tomato* DC3000. Overall, this work aims to understand the relationship between lignin cationization, antimicrobial potential, and plant compatibility and to assess the feasibility of using CL‐based hydrogels as bioactive materials for plant protection applications.

## 2 Methodology

### 2.1 Materials

Softwood kraft lignin (BioPiva 100) was obtained from UPM Biochemicals (Finland). Glycidyl trimethylammonium chloride (GTMAC), 1,4‐dioxane, genipin, chitosan (medium molecular weight, CAS Number 9012‐76‐4), glacial acetic acid, and 2‐chloro‐4,4,5,5‐tetramethyl‐1,3,2‐dioxaphospholane (TMDP) were purchased from Sigma–Aldrich (USA). Sodium hydroxide (NaOH) was purchased from Fisher Chemical (USA), and *N*,*N*‐dimethylformamide (DMF, anhydrous) was purchased from Acros Organics (USA). *N*‐Hydroxy‐5‐norbornene‐2,3‐dicarboximide (NHND) was purchased from TCI Chemicals (Japan), and chromium(III) acetylacetonate was purchased from Thermo Scientific (USA). ROTICell PBS w/o Ca and Mg and 2‐(*N*‐Morpholino)ethanesulfonic acid (MES) were purchased from Carl Roth (Germany). Luria Bertani (LB) broth, LB agar, Dulbecco’s phosphate‐buffered saline (DPBS), and glycerol were purchased from Thermo Fisher Scientific (USA). Dialysis membranes (Spectra/Por 6, MWCO 1 kDa) were obtained from Spectrum Laboratories (USA). Milli‐Q water was used throughout the experiments. All chemicals were used as received without further purification.

### 2.2 Methods

#### 2.2.1 Cationization of Lignin

The cationization of lignin was performed following a procedure previously reported in the literature [10, 11], with modifications. Kraft lignin (450 mg) was dispersed in Milli‐Q water to obtain a 3 wt% lignin solution with a total reaction volume of 15 mL. To dissolve the lignin, 1 mL of 1 M NaOH was added to the suspension under stirring to achieve a pH of 13. The reaction mixture was heated to 70 °C, after which GTMAC was added dropwise under stirring at the desired mass ratios relative to lignin. The reaction proceeded for 1 h at the specified temperature. After the reaction, the mixture was cooled to room temperature.

The product was purified by dialysis against Milli‐Q water for 72 h (MWCO ~1 kDa), with the water exchanged every 8 h. The purified solution was subsequently lyophilized for 48 h to obtain CL as a fluffy brown powder. Different DS were achieved by varying the lignin/GTMAC ratio (1:X, w/w) using ratios of 1:1, 1:2, 1:3, 1:4, and 1:5. The resulting samples were denoted as CLX, where X corresponds to the GTMAC ratio. The reaction yield was calculated from the dry mass of the CL samples after lyophilization relative to the initial mass of kraft lignin.

#### 2.2.2 Zeta Potential Measurements

The surface charge of bare lignin and CL samples was determined by zeta potential measurements. Samples were prepared in triplicate by dissolving them in 1 mL of Milli‐Q water to obtain solutions with a concentration of 1 mg mL^−1^. The solutions were sonicated for 1 min and mixed using a vortex mixer to ensure complete dissolution. The measurements were performed using a Zetasizer Ultra (Malvern, UK) at 25 °C. Prior to measurement, 750 µL of each sample solution was transferred into DTS1070 cells. For each sample, three independent replicate solutions were prepared. Each replicate solution was measured three times by the instrument, and the average of these measurements was used as one replicate value.

#### 2.2.3 Elemental Analysis

Elemental analysis was conducted to determine the carbon (C), hydrogen (H), nitrogen (N), and sulfur (S) content of bare lignin and CL samples using a Vario EL (Elementar Analysensysteme, Germany). Approximately 2 mg of each sample was used for measurement.

The DS, defined as the number of quaternary ammonium groups introduced per lignin unit, was calculated from the nitrogen content according to Equation (1), as reported in the literature [10]

(1) $$\mathrm{DS}=\frac{180.0\;\text{N\%}}{1400.0-151.5\;\text{N\%}}$$

where N% represents the nitrogen content obtained from elemental analysis. The values 180.0 and 151.5 correspond to the molar masses of the C_9_ lignin unit and GTMAC, respectively, while 1400.0 corresponds to the molar mass of nitrogen multiplied by 100.

#### 2.2.4 Determination of Phenolic Hydroxyl Content by UV–Vis Spectroscopy

The concentration of phenolic hydroxyl (OH) groups in the lignin samples was determined by UV–vis spectroscopy using a Lambda 365 (PerkinElmer, USA). Stock solutions (1 mg mL^−1^) were prepared by dissolving 5 mg of bare lignin or CL in 5 mL 1,4‐dioxane/0.2 M NaOH (1:1, v/v). The mixture was sonicated for 1 min until a homogeneous solution was obtained. UV–vis measurements were performed in three technical replicates for each sample, and the average value was used for phenolic OH quantification.

For preparation of the blank solution, 240 µL of the stock solution was mixed with 2.76 mL of 50 mM MES buffer (pH 6.0) to obtain a final lignin concentration of 0.08 g L^−1^. For measurement samples, 560 µL of the lignin stock solution was mixed with 6.44 mL of 0.2 M NaOH, yielding an alkaline solution of the same final lignin concentration (0.08 g L^−1^). The volume of the alkaline solution was prepared to allow three replicate measurements.

The concentration of the phenolic OH groups was calculated using Equation (2), as reported in the literature [39]

(2) $${\mathrm{OH}}_{\text{phenolic}}=\frac{\left[0.250\times A_{300\text{nm}}+0.107\times A_{350\text{nm}}\right]}{\mathrm{cd}}$$

where *A*_300nm_ and *A*_350nm_ correspond to the absorbance values measured at 300 and 350nm, respectively. *c* represents the concentration of the lignin (0.08 g L^−1^), whereas *d* is the path length of the light (1 cm).

#### 2.2.5 Fourier Transform Infrared Spectroscopy (FTIR)

Fourier transform infrared (FTIR) spectroscopy was performed to characterize bare lignin and CL samples. Measurements were carried out using an Alpha II FTIR spectrometer (Bruker Optik, Germany). Before analysis, the samples were lyophilized to ensure complete drying. Spectra were recorded over the range of 4000–400 cm^−1^, with 24 scans collected at a resolution of 4 cm^−1^.

#### 2.2.6 Gel Permeation Chromatography (GPC)

Gel permeation chromatography (GPC) was performed to determine the molecular weight distribution of bare lignin and CL samples. Measurements were carried out using a WinGPC UniChrom 8.2 system (PSS, Germany) equipped with an HPLC COMPACT PUMP 3350 (Bischoff, Germany). DMSO containing 0.075 M NaNO_3_ was used as the eluent at a flow rate of 0.5 mL min^−1^, and the columns were maintained at 60 °C. Detection was performed using a refractive index detector (Shodex RI‐501, Showa Denko, Japan) and a photodiode array detector (SPD‐M20A, Shimadzu, Japan) operating at 280 nm. Calibration was carried out using pullulan standards (PSS, Germany). Prior to analysis, sample solutions (2–3 mg mL^−1^) were filtered through 0.2 µm syringe filters.

#### 2.2.7 ^31^P Nuclear Magnetic Resonance (NMR) Analysis of Hydroxyl Groups

^31^P nuclear magnetic resonance (NMR) analysis of OH groups was performed based on previous work [40]. Bare lignin or CL samples were dried in an oven at 45 °C overnight and cooled to room temperature in a vacuum desiccator. Approximately 30 mg of each dried sample was dissolved in 0.6 mL of an anhydrous CDCl_3_/pyridine mixture (1:1.6, v/v).

An internal standard (IS) solution containing N‐hydroxy‐5‐norbornene‐2,3‐dicarboximide (NHND, 18 mg mL^−1^) and chromium(III) acetylacetonate (Cr(acac)_3_, 5 mg mL^−1^) was prepared in the same CDCl_3_/pyridine mixture. Approximately 0.1 mL of this IS solution was added to the sample, and the mixture was stirred overnight. Subsequently, 2‐chloro‐4,4,5,5‐tetramethyl‐1,3,2‐dioxaphospholane (TMDP) was added. If required, up to 0.1 mL of anhydrous DMF was added to improve dissolution. The exact masses of all added components were recorded.

^31^P NMR spectra were recorded on an ECX400 (JEOL, Japan) spectrometer operating at a proton resonance frequency of 400 MHz, equipped with an automatically tunable probe. Spectra were processed using MestReNova (Mestrelab Research, Spain).

#### 2.2.8 Thermogravimetric Analysis

Thermogravimetric analysis (TGA) was performed using a TGA 8000 (PerkinElmer, USA) to evaluate the thermal stability of bare lignin and CL samples. Approximately 5 mg of each sample was analyzed under a nitrogen atmosphere (40 mL min^−1^). Samples were initially held at 30 °C for 1 min for stabilization, followed by heating to 800 °C at a rate of 20 °C min^−1^. After the heating cycle, the system was cooled to 30 °C at 100 °C min^−1^. The results are expressed as mass loss (%) relative to the initial sample mass.

#### 2.2.9 Phytotoxicity Assays

Phytotoxicity assays were performed to evaluate the compatibility of CL samples with plant tissue by infiltrating 4–6‐week‐old *Nicotiana benthamiana* leaves, adapted from previously described leaf infiltration procedures [41, 42, 43]. Briefly, the sample solutions of CL were dissolved in a 10 mM MgCl_2_ and 10 mM MES buffer solution at concentrations of 0.1, 0.5, 1.0, 1.5, and 2.0 mg mL^−1^. Leaf infiltrations were carried out on the abaxial (bottom) side of the leaf using a needleless syringe, with ~500 µL of solution injected per infiltration site. Each leaf was infiltrated at four different spots, consisting of three replicate infiltrations of the same sample concentration and one blank control spot containing the MgCl_2_ + MES buffer solution. Each condition was tested in three independent infiltration experiments.

While still attached to the plant, the infiltrated leaves were monitored for 5 days for any visible signs of necrosis or tissue damage. At 5 days post‐infiltration (dpi), the leaves were detached and imaged using a digital camera for visible‐light images and a ChemiDoc MP Imaging System (Bio‐Rad, USA) for infrared imaging. Infrared imaging was performed using a multichannel mode, including green (IRDye 680RD Blot), red (DyLight 800 Blot), and blue (colorimetric blot) channels. Raw images were processed using Fiji (ImageJ distribution) [44], where the blue channel was removed to enhance the view of the tissue damage shown as red, based on previously described protocols [45].

#### 2.2.10 Electrolyte Leakage Assay

Electrolyte leakage was measured as a quantitative indicator of membrane damage in *N. benthamiana* leaf tissue after infiltration with CL solutions, adapted from previously reported methods [46, 47, 48]. CL1, CL2, and CL3 solutions were tested at concentrations of 0.1, 0.5, 1.0, 1.5, and 2.0 mg mL^−1^ in 10 mM MgCl_2_ + 10 mM MES buffer. Leaves infiltrated with the same 10 mM MgCl_2_ + 10 mM MES buffer without CL were used as the control. At 5 dpi, 10 mm leaf disks were excised from the infiltrated regions using a cork borer and placed in Milli‐Q water for 30 min to remove electrolytes released due to tissue excision. The water was then discarded, and the leaf disks were transferred into fresh Milli‐Q water. Conductivity was measured in µS cm^−^¹ using an EC‐3 conductivity meter (HM Digital, USA) immediately after transfer into fresh water to record the baseline conductivity and again after 4 h of incubation at room temperature. The samples were then heated inside the tubes in a water bath at 90 °C for 45 min to disrupt the leaf tissue and release total cellular electrolytes, allowing the maximum conductivity to be determined after cooling to room temperature. Electrolyte leakage was calculated as the percentage of conductivity after 4 h relative to the maximum conductivity after heat treatment using Equation (3)

(3) $$\text{Electrolyte leakage}\;(\%)=\frac{C_{4h}}{C_{\max}}\times 100$$

where *C*_4h_ is the conductivity after 4 h of incubation and *C*_max_ is the maximum conductivity measured after heat treatment and cooling. This calculation normalizes the 4 h conductivity value to the total releasable electrolyte content of each leaf disk, thereby accounting for biological variation between leaf samples. Each condition was analyzed using three independent biological replicates.

#### 2.2.11 Synthesis of Chitosan–Genipin Hydrogels

CH–GPN hydrogels were prepared using a solution‐based cross‐linking approach. Chitosan was first dissolved in 1% (v/v) aqueous acetic acid to obtain a 1.5% (w/v) solution, which was stirred for 20 h until a homogeneous, viscous solution was obtained. The pH of the chitosan solution was adjusted to ~5.5 by adding 1 M NaOH. Genipin was dissolved in Milli‐Q water to prepare a fresh aqueous stock solution with a concentration of 0.1% (w/v). 6 mL of chitosan solution was mixed with the genipin solution to obtain a GPN/CH ratio of 0.25% (w/w). The final volume was adjusted to 7 mL with Milli‐Q water. For CL‐containing hydrogels, CL2 was used as the representative CL due to its strong antibacterial activity and good aqueous processability. CL2 was added to the reaction mix as an aqueous stock solution (20 mg mL^−1^) prior to the addition of genipin. The CL2 content was varied from 1 to 20 wt%, calculated relative to the chitosan mass, while the chitosan mass, GPN/CH ratio, and final formulation volume were kept constant. The resulting mixtures were stirred for 10 min to ensure homogeneous distribution of all components. After mixing, the solutions were transferred into vials and cured at 37 °C for 18 h to allow gelation and network formation. Hydrogels synthesized without CL2 were cured using the same procedure and served as control samples. After curing, gelation was confirmed by turning the vial upside down, where the hydrogels retained their shape without water‐like flow. Additionally, a visible color change was observed, which was associated with genipin‐mediated cross‐linking of chitosan.

#### 2.2.12 Rheological Characterization of Hydrogels

The viscoelastic properties of the hydrogels were characterized using a Kinexus (Malvern, UK) rotational rheometer equipped with a plate–plate geometry (25 mm diameter). Measurements were performed at 10 and 25 °C to compare hydrogel behavior under lower‐temperature and room‐temperature conditions, respectively. Frequency sweep experiments were conducted over a range of 0.1–10 Hz at a constant strain of 0.5% within the low‐strain viscoelastic region (Figure S10). The storage modulus (*G*′), loss modulus (*G*″), and complex viscosity were recorded to evaluate the mechanical properties and network integrity of the hydrogels.

#### 2.2.13 Scanning Electron Microscopy Imaging of Hydrogels

Scanning electron microscopy (SEM) analysis of the hydrogel samples was performed using a Sigma 300 VP field emission scanning electron microscope (Zeiss, Germany). Hydrogels were prepared 24 h prior to analysis. For internal morphology characterization, the hydrogels were freeze‐dried and sectioned into thin slices using a clean blade. The dried samples were mounted onto coverslips using a thin carbon/chromium coating to improve sample adhesion and reduce charging effects during imaging. The samples were subsequently gold‐coated and dried at room temperature (25 °C) prior to SEM analysis.

#### 2.2.14 CL2 Release from CH–GPN–CL2 Hydrogels

CL2 release was evaluated using an adapted lignin‐based hydrogel release setup [49]. Briefly, 250 mg of wet CH–GPN–CL2 hydrogel was immersed in 10 mL of a release medium containing 10 mM MES and 10 mM MgCl_2_. CH–GPN–CL2 hydrogels containing 1, 2.5, 5, 10, 15, and 20 wt% CL2 were tested. Samples were incubated at room temperature. At 0.5, 1, 2, 3, 4, and 5 h, 2 mL of supernatant was collected and replaced with 2 mL of fresh release medium. Before sampling, the samples were gently tilted to homogenize the medium while minimizing hydrogel disruption. The collected aliquots were centrifuged at 5000 × *g* for 5 min at room temperature to remove hydrogel fragments, and the clarified supernatants were analyzed at 280 nm using a Lambda 365 UV–vis spectrophotometer (PerkinElmer, USA). For each hydrogel formulation, one wet hydrogel sample was tested. Each collected aliquot was measured three times by UV–vis spectroscopy, and the average absorbance was used for the CL2 concentration calculation. These repeated measurements were technical measurements of the same aliquot, not independent experimental replicates. The release medium alone was used as the instrument baseline. The wavelength of 280 nm was selected based on previous UV–vis quantification of lignin‐based compounds [50, 51, 52].

CL2 concentrations were estimated from a CL2 standard curve prepared in the same MES + MgCl_2_ buffer using concentrations of 1, 2.5, 5, 10, 25, 50, 75, and 100 µg mL^−1^ (Figure S14). Supernatants from CH–GPN hydrogels without CL2 were measured under the same conditions, and their absorbance was subtracted from the absorbance of the corresponding CH–GPN–CL2 hydrogel samples for blank correction. For each time point, blank correction was performed using the average absorbance values of the CH–GPN and CH–GPN–CL2 hydrogel supernatants.

Cumulative CL2 release was calculated by accounting for the CL2 present in the release medium at each time point and the CL2 removed in previous aliquots, as described in Equation (S1). Here, cumulative release refers to the total estimated amount of CL2 released up to a given time point, including both the CL2 remaining in the release medium and the CL2 removed during previous sampling steps. Results were reported as estimated cumulative CL2 release per 250 mg wet hydrogel.

#### 2.2.15 Water Uptake and Swelling Capacity of CH–GPN–CL2 Hydrogels

Water uptake and swelling capacity were evaluated using lyophilized CH–GPN and CH–GPN–CL2 hydrogel pieces. Hydrogel pieces with an initial dry mass of approximately 4 mg were individually immersed in 20 mL Milli‐Q water at room temperature. At 10, 20, 30, and 40 min, the swollen hydrogel was removed from the water, excess surface water was gently removed, and the wet mass was recorded. Water uptake was calculated as *m*_t_–*m*_0_, where *m*_0_ is the initial dry mass and *m*_t_ is the swollen mass at each time point. The swelling ratio was calculated as (*m*_t_–*m*_0_)/*m*_0_.

#### 2.2.16 Bacterial Inhibition Assay

*Pseudomonas syringae* pv. *tomato* DC3000 was streaked onto LB agar plates from a frozen glycerol stock solution and incubated at 28 °C. A single colony was used to inoculate LB broth and was cultured at 28 °C with orbital shaking at 200 rpm until an optical density at 600 nm (OD_600_) of approximately 0.7 was reached. The culture was subsequently diluted in fresh LB medium to obtain a working inoculum of approximately 1 × 10^7^ CFU mL^−1^, which was verified by serial dilution and colony enumeration on LB agar plates. Subsequently, 5 μL of this culture was added to 200 μL of UV‐sterilized CL samples or hydrogels of varying concentrations in a sterile 96‐well round‐bottom plate (Sarstedt). For the free CL assay, bare lignin and CL1–CL3 were tested at concentrations of 5–500 μg mL^−1^. For the hydrogel‐based assay, the tested samples were the CH–GPN control hydrogel (without CL2) and CH–GPN–CL2 hydrogels containing 1, 2.5, 5, 10, and 15 wt% CL2. These hydrogels were prepared using the optimized GPN/CH ratio of 0.25% (w/w).

The contents of each well were mixed thoroughly by pipetting. Untreated bacterial cultures served as growth controls, while sterile CL dispersions or hydrogel samples without bacteria were used as blanks. The 96‐well plate was closed with a lid and was incubated for 20 h at 28 °C with continuous double‐orbital shaking.

To determine colony forming units (CFUs), samples were collected after 20 h exposure to the CL/hydrogel materials and serially diluted in DPBS. From each dilution, 100 μL was transferred onto LB agar plates and allowed to dry completely in a clean bench hood. Plates were incubated for 2 days at 28 °C, after which colonies were counted and compared with the untreated control. The CFU mL^−1^ were back‐calculated based on the plated volume and the dilution factor. The limit of detection (LOD) of the CFU assay was 30 CFU mL^−1^, calculated using a plated volume of 100 μL. Samples yielding no detectable colonies were reported as below the detection limit (<LOD). For each condition, three independent biological replicates were performed, each consisting of three technical replicates.

The same hydrogel‐based CFU assay was also performed using the beneficial plant‐associated bacterium *Sphingomonas melonis* Fr1 under the same exposure and plating conditions, unless otherwise stated.

To evaluate antibacterial activity after repeated wetting/drying, CH–GPN and CH–GPN–CL2 hydrogels were subjected to two drying–rewetting cycles before CFU analysis. Briefly, 0.200 mL of each hydrogel formulation was dried at 60 °C for 24 h with the microtube lid open, followed by rehydration in 0.3 mL deionized water at room temperature for 24 h. This cycle was repeated twice, and the samples were then tested against *P. syringae* pv. *tomato* DC3000 using the hydrogel‐based CFU assay described above.

#### 2.2.17 Live/Dead Fluorescence Staining of Treated Bacteria

Bacterial viability was evaluated using the LIVE/DEAD BacLight Bacterial Viability Kit (Invitrogen, cat# L7012) according to the manufacturer’s instructions. Briefly, after a 20 h incubation with the CL/hydrogel materials, 0.5 µL of SYTO 9 and 0.5 µL of propidium iodide (PI) were added to each well containing 200 µL of bacterial suspension. The samples were mixed via pipetting and incubated at room temperature in the dark for 15 min. A 10‐µL aliquot of the stained sample was transferred to a microscope slide and secured with a poly‐L‐lysine‐coated coverslip. The samples were imaged using a 100X oil immersion objective on a ZEISS Axio Observer. Z1 fluorescence microscope equipped with GFP (for SYTO 9) and Texas Red (for PI) filter channels. Image processing was performed using ZEISS ZEN software.

#### 2.2.18 Scanning Electron Microscopy Imaging of Treated Bacteria

Bacterial suspensions of *P. syringae* pv. *tomato* DC3000 were prepared in ROTICell PBS w/o Ca and Mg and incubated with the samples (CL and hydrogels) overnight under gentle shaking conditions. After incubation, the samples were gently washed with the PBS to remove loosely associated bacterial cells and subsequently fixed with 2.5% (v/v) glutaraldehyde for 2 h at 4 °C. The fixed samples were then washed again with PBS and dehydrated using a graded ethanol series of 30%, 50%, 70%, 90%, and 100%. The dehydrated bacterial samples were drop‐cast onto conductive carbon tape, sputter‐coated with a 10‐nm gold layer, and analyzed by SEM at a voltage of 15 kV at various magnifications.

## 3 Results and Discussion

### 3.1 Synthesis of Cationic Lignin

To explore the potential of lignin as a tunable bio‐based material, (bare) kraft lignin was functionalized with glycidyl trimethylammonium chloride (GTMAC) to introduce quaternary ammonium groups onto the lignin backbone. This reaction, as shown in Figure 1a, introduces a permanent positive charge to the lignin backbone that results in the synthesis of cationic lignin (CL). The reaction was carried out under alkaline conditions in order to deprotonate the phenolic hydroxyl (OH) groups of lignin, to obtain the reactive phenolate species required for the reaction.

**FIGURE 1:**
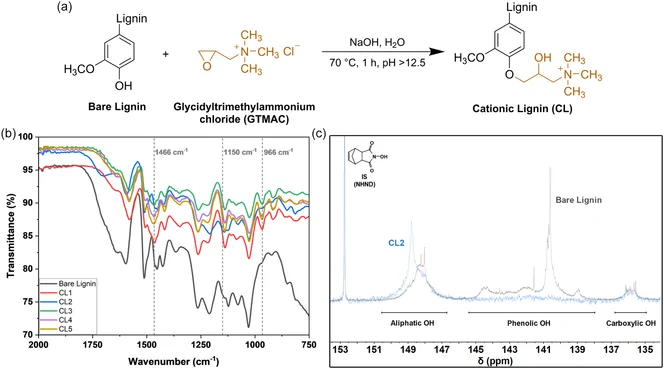
(a) Cationization of bare lignin via reaction with GTMAC under alkaline aqueous conditions, resulting in CL. (b) Enlarged FTIR spectral region of bare lignin and CL samples (CL1–CL5), highlighting characteristic bands associated with quaternary ammonium modification at 1466, 1150, and 966 cm^−1^. (c) Comparison of ^31^P NMR spectra of bare lignin and the representative CL2 after phosphitylation with TMDP, showing the characteristic regions corresponding to aliphatic, phenolic, and carboxylic hydroxyl (OH) groups. IS denotes the IS solution, which is set as the reference. Full FTIR and ^31^P NMR spectra of all CL samples are provided in the supporting information.

The cationization was performed under relatively mild conditions at 70 °C in aqueous solution. Under these conditions, the phenolate groups can react with the epoxide group of GTMAC via nucleophilic ring opening, forming a new ether bond between the lignin backbone and the quaternary ammonium group. Previous studies have shown that temperatures above ~70 °C or reaction times exceeding ~1 h can lead to increased degradation and lower yields of the functionalized lignin [11]. Therefore, the chosen reaction conditions help minimize side reactions while maintaining reactivity. In addition, the reaction and subsequent purification were carried out in aqueous media, with dialysis performed using Milli‐Q water, which supports a sustainable approach by avoiding the need for organic solvents.

A library of CL was prepared by varying the lignin/GTMAC mass ratio. Ochi et al. reported ratios of 1:2, 1:3.5, and 1:5 (w/w), which resulted in materials with different DS [53]. In this work, the range was expanded to 1:1–1:5 to cover low, medium, and high GTMAC feed ratios and to evaluate how the reagent excess influences nitrogen incorporation, DS, surface charge, and antimicrobial activity. This range also allowed us to identify a representative CL sample with sufficient cationic modification while also avoiding unnecessary use of excess GTMAC. The resulting products were denoted CLX, where X corresponds to the GTMAC ratio used in the synthesis (e.g., CL3 represents a 1:3 lignin/GTMAC mass ratio). In comparison with bare lignin, CL has a significantly better solubility in water, consistent with the literature [54]. The reaction yields after dialysis and lyophilization ranged from 59.39% to 66.53% across the CL series, with the full yield values shown in Table S1. These values indicate that CL could be recovered after purification for all tested lignin/GTMAC ratios under the applied aqueous reaction conditions.

By varying the lignin/GTMAC mass ratio, a library of CL with different DS was obtained. The resulting CL samples were characterized to analyze changes in functional groups, molecular weight distribution, and surface charge.

### 3.2 Characterization of Cationic Lignin

To confirm the successful incorporation of quaternary ammonium groups into the lignin backbone, the CL samples were analyzed by several techniques.

FTIR spectroscopy was first used to qualitatively confirm the cationization of lignin (Figure 1b), while the full spectra are shown in Figure S1. The bare lignin in this work exhibits characteristic bands typical of kraft lignin, consistent with reported assignments for lignin functional groups by Klapiszewski et al. [55]. Upon cationization, the CL samples (CL1–CL5) show a reduced intensity of the O─H band, indicating some consumption of the OH groups. In addition, new bands appear in the fingerprint region, most notably at ~1150 cm^−1^, which can be attributed to C─O─C stretching of phenolic ether linkages formed between lignin and the quaternary ammonium group as a result of cationization. The bands at ~1466 and ~966 cm^−1^ are assigned to the CH_2_ and CH_3_ groups of GTMAC, respectively [53]. This provides further evidence for successful lignin cationization, as these features became more pronounced in the CL samples and are consistent with previous reports on cationization of lignin with GTMAC [14, 15].

Elemental analysis was used to determine the extent of the cationization of lignin, since the introduction of quaternary ammonium groups introduces nitrogen into its structure. In contrast to (bare) kraft lignin, which contains little to negligible amounts of nitrogen [56], all CL samples show increased nitrogen content after cationization across all the different lignin/GTMAC ratios (Table 1). The same is true for DS, as it was calculated using Equation (1) from the nitrogen content obtained via elemental analysis. As expected with increasing GTMAC ratios, the nitrogen content and DS increased from CL1 to CL3, indicating a higher incorporation of quaternary ammonium groups. However, this increase becomes less pronounced at higher ratios, with CL5 showing only a slight change compared to CL4. The complete elemental analysis data are shown in Table S1.

**TABLE 1:** Physicochemical characterization of bare lignin and CL samples prepared at different lignin/GTMAC mass ratios. Zeta potential, nitrogen content, DS, phenolic hydroxyl content, and weight‐average molecular weight (*M*_w_) are summarized.

Sample	Lignin/GTMAC mass ratio, w/w	Zeta potential, mV	*N*, %^a^	DS, %^b^	Phenolic OH, mmol g^−1^ c	*M*_w_, kDa^d^
Bare lignin	—	−42.80	0.23	—	1.0096	2.823
CL1	1:1	24.80	2.41	41.92	0.3354	6.007
CL2	1:2	31.95	2.60	46.52	0.2771	7.671
CL3	1:3	39.22	3.00	57.11	0.1980	8.044
CL4	1:4	34.24	3.16	61.74	0.1452	8.471
CL5	1:5	40.99	3.06	58.82	0.1520	8.059

a Determined by elemental analysis.

b Calculated from elemental analysis using Equation (1).

c Calculated from UV–vis spectroscopy using Equation (2).

d Determined by GPC.

This observation was further supported by the phenolic OH content determined by UV–vis spectroscopy. After cationization, the phenolic OH content decreased for all CL samples, which is consistent with the involvement of lignin hydroxyl groups in the nucleophilic ring opening of the GTMAC epoxide group. This trend is also relevant to the lignin source used in this work. BioPiva 100, the kraft lignin used in this study, is a softwood kraft lignin and is therefore expected to be mainly composed of guaiacyl (G) units [57, 58, 59]. It contains reactive functional groups, including phenolic and aliphatic OH groups, methoxy groups, and ether linkages that influence lignin reactivity during chemical modification. Under alkaline conditions, accessible phenolic OH groups can be deprotonated and react with GTMAC, supporting the observed decrease in phenolic OH content after cationization. Across the CL series, this decrease became less pronounced at higher GTMAC ratios. Since UV–vis spectroscopy provides an indirect estimation of phenolic OH content based on absorbance changes under alkaline conditions, these values are interpreted primarily in terms of relative trends across the CL series rather than as absolute OH group concentrations.

The increase in nitrogen content is also reflected in the average zeta potential measurements shown in Table 1. Bare lignin exhibits a negative surface charge (−42.8 mV) consistent with the negative values reported in the literature [55]. The CL samples display positive zeta potentials, ranging from 24.8 mV for CL1 to 40.99 mV for CL5. It is also notable that a complete charge reversal from negative to positive values is observed after cationization. This shift in surface charge indicates the successful introduction of quaternary ammonium groups onto the lignin backbone, as these permanently charged groups dominate the surface properties of CL. The full zeta potential distributions and corresponding average values are shown in Figure S2, further illustrating the shift toward positive surface charge upon modification.

Overall, the characterization results confirm the successful cationization of lignin. As shown in Table 1, a common trend can be observed across the CL series: The increase in nitrogen content and DS, together with the loss of phenolic OH groups, becomes less pronounced at higher lignin/GTMAC ratios, particularly between CL4 and CL5. This suggests that the reaction approaches a plateau‐like saturation effect, likely due to the reduced availability of accessible OH groups and/or steric hindrance at higher GTMAC feed ratios. In addition, zeta potential measurements confirm the charge reversal from negatively charged bare lignin to positively charged CL samples. This overall trend is further visualized in the normalized comparison of the quantified characterization data shown in Figure S3, which compares the normalized changes in zeta potential, DS, *M*_w_, and phenolic OH loss across CL1–CL5 and highlights the reduced change between the higher GTMAC feed ratios, particularly CL4 and CL5.

### 3.3 Additional Structural Characterization of Cationic Lignin

GPC was used to evaluate changes in the molecular weight distribution of lignin after cationization (Figure S4). The *M*_w_ values discussed here refer only to bare lignin and free CL samples before hydrogel incorporation and not to the CH–GPN–CL hydrogel formulations. Bare lignin exhibits a broad molecular weight distribution due to its heterogeneous structure, whereas all CL samples display narrower and more defined peaks with increasing DS. In addition, a shift toward higher molecular weight is observed for the CL samples, reflecting the incorporation of GTMAC moieties. The increase in *M*_w_ follows the same trend as the DS values obtained from elemental analysis (Table 1), further supporting successful lignin functionalization. Since GPC was performed once per sample, the *M*_w_ values are used mainly to compare relative changes across the CL series rather than as statistically averaged values. The *M*_w_ increase also became less pronounced at higher lignin/GTMAC ratios, in agreement with the saturation effect discussed above.

^31^P NMR analysis of OH groups further supports the successful modification of lignin via quantification of the concentration of different OH groups after cationization. During sample preparation, small amounts of anhydrous DMF were added to all CL samples due to solubility issues in the NMR solvent. Despite this, all CL samples show relatively low signal‐to‐noise ratios when compared to bare lignin, which limits the reliability of this technique in quantification. A representative comparison between bare lignin and CL2 (Figure 1c) shows that the characteristic phenolic OH signals (145–138 ppm), which are typical in lignin according to literature reports [14, 40], are strongly suppressed after cationization. On the other hand, the aliphatic OH signals (149–147 ppm) remain visible and appear slightly shifted, consistent with previous reports in the literature [14]. This may be related to the newly formed secondary aliphatic OH group introduced during GTMAC ring opening, while the reaction still preferentially consumes phenolic OH groups. This is true across all CL samples, of which the spectra are shown in Figure S5. The quantified phenolic and aliphatic OH group concentrations are shown in Table S2. Similar to the trends shown in Table 1, the phenolic OH concentration decreases significantly after cationization and again shows a saturation effect between CL4 and CL5. Furthermore, no clear trend is observable for the aliphatic OH concentrations, suggesting that GTMAC at higher ratios does not considerably react with aliphatic OH groups. Similar to the UV–vis results, the ^31^P NMR data show a clear decrease in phenolic OH concentration after cationization and a less pronounced change between CL4 and CL5. However, the absolute phenolic OH values obtained by UV–vis and ^31^P NMR differ, likely due to the different sample preparation conditions and detection principles of the two methods. Therefore, both datasets are used here mainly to compare relative trends rather than direct agreement in absolute values. Additionally, due to the noisy spectra caused by poor solubility of the CL samples during phosphitylation, the ^31^P NMR results should be interpreted cautiously.

TGA shows that the overall thermal degradation behavior of lignin is largely retained after cationization (Figure S6). In agreement with Ochi et al. [53], both bare lignin and CL samples exhibit multistep degradation, with an initial weight loss below 200 °C attributed to water desorption. Compared to bare lignin, the CL samples display reduced thermal stability initially, indicated by a higher rate of mass loss around ~260 °C, which is attributed to the decomposition of quaternary ammonium groups. This is followed by the main decomposition of the lignin backbone in the range of 400–500 °C. In contrast, bare lignin shows a more gradual mass loss profile over these temperature ranges. Additionally, most samples do not fully degrade up to 800 °C under inert atmosphere and instead leave a residual char. This is consistent with the literature, where lignin‐based materials form carbon‐rich residues under inert conditions [60, 61], while complete degradation is only observed in oxidative environments due to combustion [60]. A slight decrease in thermal stability is observed for CL5, which may be attributed to the higher content of quaternary ammonium groups introducing thermally less stable aliphatic functionalities.

Overall, these results are in good agreement with the trends obtained from elemental analysis, UV–vis spectroscopy, and zeta potential measurements and collectively confirm the successful cationization of lignin while preserving its overall structural framework.

### 3.4 Plant Compatibility of Cationic Lignin in *Nicotiana benthamiana* Leaves

Phytotoxicity assays are important for evaluating whether introduced treatments cause toxic effects in plant tissues before further in planta testing. For this purpose, leaf infiltration assays are widely used in plant science for transient protein expression studies or to study plant responses to externally introduced compounds under controlled conditions [42]. In particular, the infiltration of *Nicotiana benthamiana* leaves allows for localized delivery of materials directly into the leaf tissue, making it a useful method for evaluating plant responses [36, 37, 38, 42]. Therefore, leaf infiltration assays were performed to evaluate the plant compatibility of CL. A representative experimental setup is shown in Figure 2a, where R1–R3 indicate replicate infiltration spots for the same CL sample and concentration. A visible‐light image of infiltrated leaves and the corresponding infrared image are shown in Figure 2b,c, respectively. Electrolyte leakage was additionally measured as a quantitative indicator of membrane damage and is shown in Figure 2d. The complete imaging dataset for all tested samples (CL1, CL2, and CL3) and concentrations (0.1‍–‍2.0 mg mL^−1^) is shown in Figures S7–S9, with each condition tested in three independent infiltration experiments. Only CL1‍–‍CL3 were included in these assays, as higher substitution levels (CL4 and CL5) did not show significant differences from CL3 in DS due to the previously discussed saturation effect.

**FIGURE 2:**
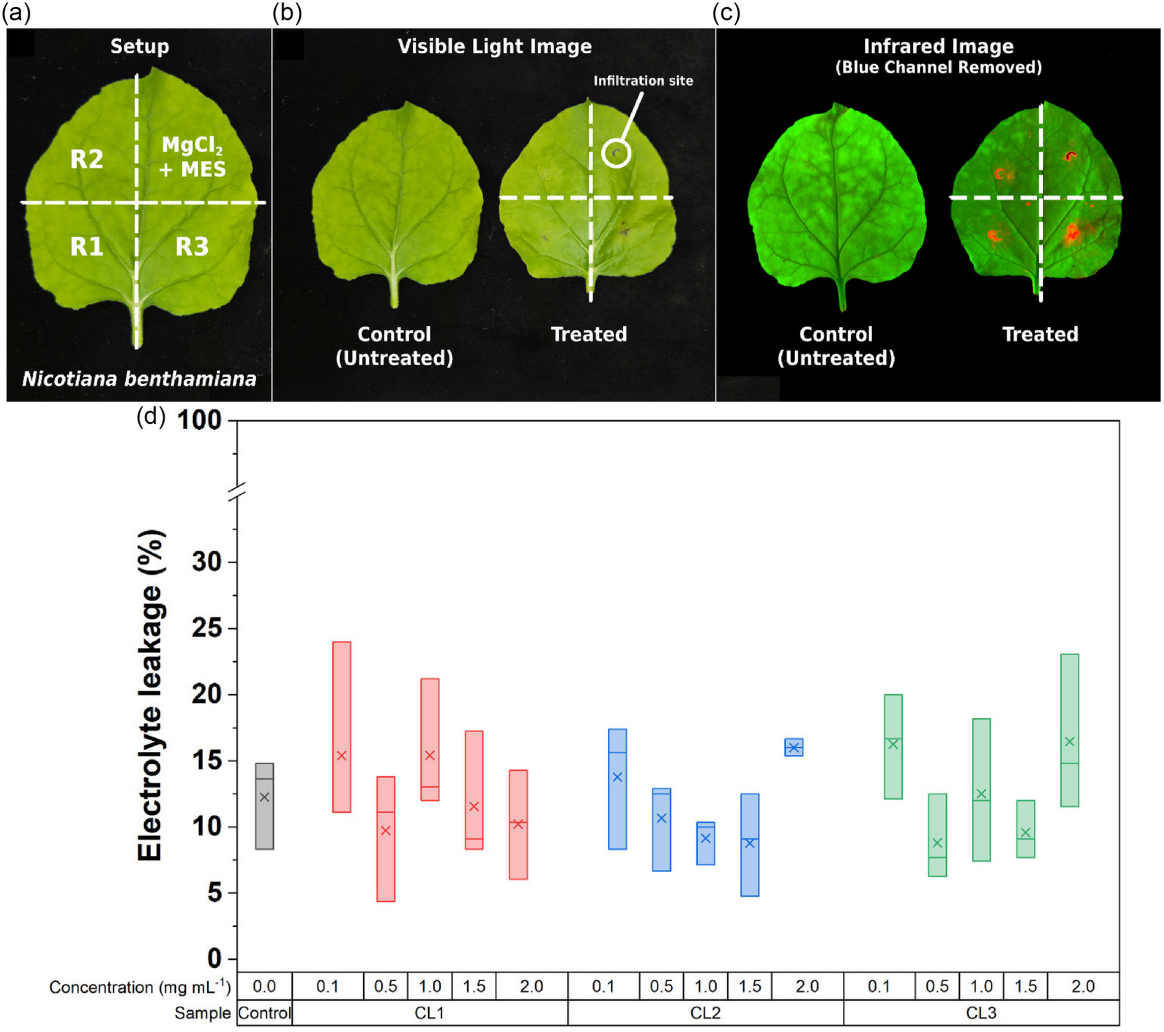
Phytotoxicity and electrolyte leakage analysis of *Nicotiana benthamiana* leaves after CL infiltration. (a) Infiltration setup showing three replicate sample spots (R1–R3) and one mock control spot containing 10 mM MgCl_2_ + 10 mM MES buffer. (b) Representative visible‐light image of CL2 at 1.0 mg mL^−1^, with the left leaf as the untreated control and the right leaf as the treated sample. (c) Corresponding infrared image, with the blue channel removed, highlighting stress‐associated signals at the injection sites. (d) Electrolyte leakage from leaf disks collected 5 days post‐infiltration (5 dpi) with CL1, CL2, or CL3 at different concentrations in 10 mM MgCl_2_ + 10 mM MES buffer. The buffer‐only infiltration was used as the control. Data are shown as box plots from three independent biological replicates (*n* = 3), with the horizontal line indicating the median and the X indicating the mean.

Leaf infiltration was performed using CL samples dissolved in infiltration buffer (10 mM MgCl_2_ + 10 mM MES), with the buffer serving as the mock control. This method is commonly used for leaf infiltration assays [42, 62]. Under these conditions, no discernible concentration‐dependent or DS‐dependent phytotoxic effects were observed. The infrared signal, where red coloration indicates stress/damage in the leaf tissue, was limited to the injection sites, suggesting that the observed damage is primarily caused by mechanical stress from infiltration using the needleless syringe. Beyond the infiltration sites, the treated tissue at R1‍–‍R3 did not react differently to the buffer control. In typical *N. benthamiana* infiltration assays, strong and widespread cell death can be induced by biological triggers such as pathogen effectors or viral proteins, leading to chlorosis, necrosis, or even tissue collapse across the infiltrated area [45, 63]. This was not observed here, as the response was confined to the injection sites, indicating that it is caused by mechanical stress during infiltration rather than the toxicity of the samples.

To quantify whether CL infiltration caused potential membrane damage, electrolyte leakage was measured from leaf disks collected from the infiltrated regions at 5 dpi. Briefly, electrolyte leakage was determined from conductivity values recorded in µS cm^−^¹ for leaf disks infiltrated with CL1, CL2, or CL3 at 0.1–2.0 mg mL^−1^ in 10 mM MgCl_2_ + 10 mM MES buffer, with buffer‐only infiltration used as the control. Since loss of membrane integrity during cell damage or cell death leads to the release of cellular electrolytes, higher electrolyte leakage values indicate stronger tissue damage [48, 64]. The electrolyte leakage values remained comparable to the MgCl_2_ + MES buffer control across all CL samples and concentrations, ranging from 8.78 ± 3.88% to 16.48 ± 5.95%, compared with 12.26 ± 3.45% for the buffer control (Figure 2d). The corresponding conductivity values and calculated electrolyte leakage values are shown in Table S3. One‐way ANOVA showed no significant differences among the MgCl_2_ + MES buffer control and CL‐treated groups (p=0.262; Table S4), indicating that CL infiltration did not significantly increase electrolyte leakage under the tested conditions. Within the tested concentration range, the electrolyte leakage values did not show a clear concentration‐dependent increase for CL1, CL2, or CL3. Similarly, increasing DS did not lead to a consistent increase in electrolyte leakage, indicating that higher DS did not translate into stronger membrane damage under the tested conditions.

Overall, the combined imaging and electrolyte leakage results indicate that the buffered CL solutions were well tolerated by *N. benthamiana* leaf tissue under the tested conditions, with no substantial membrane damage compared with the buffer control. These findings support the compatibility of CL after leaf infiltration. Since residual free GTMAC, if present, could contribute to potential toxicity, the CL samples were purified by dialysis before plant testing. After purification, CL itself represents the relevant cationic bioactive material to assess for possible plant tissue effects. The compatibility testing in this study therefore focused on CL before its incorporation into the CH–GPN hydrogel matrix. Chitosan has been widely explored in plant protection applications and described as a biodegradable and biocompatible material with roles as a plant defense elicitor, antimicrobial agent, and plant growth‐promoting material [65, 66]. Genipin is a plant‐derived cross‐linker commonly used as a biocompatible, low‐cytotoxicity alternative to conventional cross‐linkers [67]. Therefore, separate compatibility testing of CH and GPN alone was not prioritized in the present study. However, future studies should evaluate the plant compatibility of the complete CH–GPN–CL hydrogel formulation under application‐relevant conditions, such as leaf surface coating or sprayable formulations, and include an appropriate positive control for cell death to benchmark the electrolyte leakage response.

### 3.5 Hydrogel Synthesis and Composition

CH–GPN hydrogels were first optimized by varying the genipin‐to‐chitosan ratio while keeping the chitosan amount constant. The tested formulations are shown in Table S5. Formulations containing ≤0.10% GPN/CH did not form stable gels, indicating that the genipin content was too low to produce sufficient cross‐linking under the applied curing conditions. Gelation was observed from 0.15% GPN/CH onward. However, the 0.15% and 0.20% formulations showed lower mechanical stability compared to the 0.25% formulation, as indicated by the storage modulus obtained from rheological analysis (Figure S11a). This confirms that increasing the genipin content improved the stability of the CH–GPN network, although higher genipin amounts were avoided to minimize excess use of the expensive cross‐linker. Therefore, a GPN/CH ratio of 0.25% was selected for further hydrogel preparation, as it provided sufficient stable gelation.

Based on this optimized formulation, CL‐embedded hydrogels were prepared using CL2 as a representative CL sample. CL2 was selected because it provides sufficient cationic modification while requiring a lower lignin/GTMAC mass ratio than the more highly modified CL samples. It therefore represents a practical balance between functionalization degree and reagent use.

The CH–GPN hydrogel network is formed through the reaction between genipin and the primary amine groups of chitosan, resulting in CH–GPN cross‐linking (Figure 3a). In contrast, CL does not contain primary amine groups and is therefore not expected to directly participate in the genipin‐mediated cross‐linking reaction. Instead, CL is considered to be physically retained within the CH–GPN matrix. This resembles a semi‐interpenetrating polymer network (semi‐IPN)‐like system in which a cross‐linked polymer network is penetrated by linear or branched polymers without covalent incorporation between the components, according to the IUPAC definition [68, 69]. In this system, the CH–GPN phase forms the covalently cross‐linked network, while CL is physically embedded rather than covalently incorporated.

**FIGURE 3:**
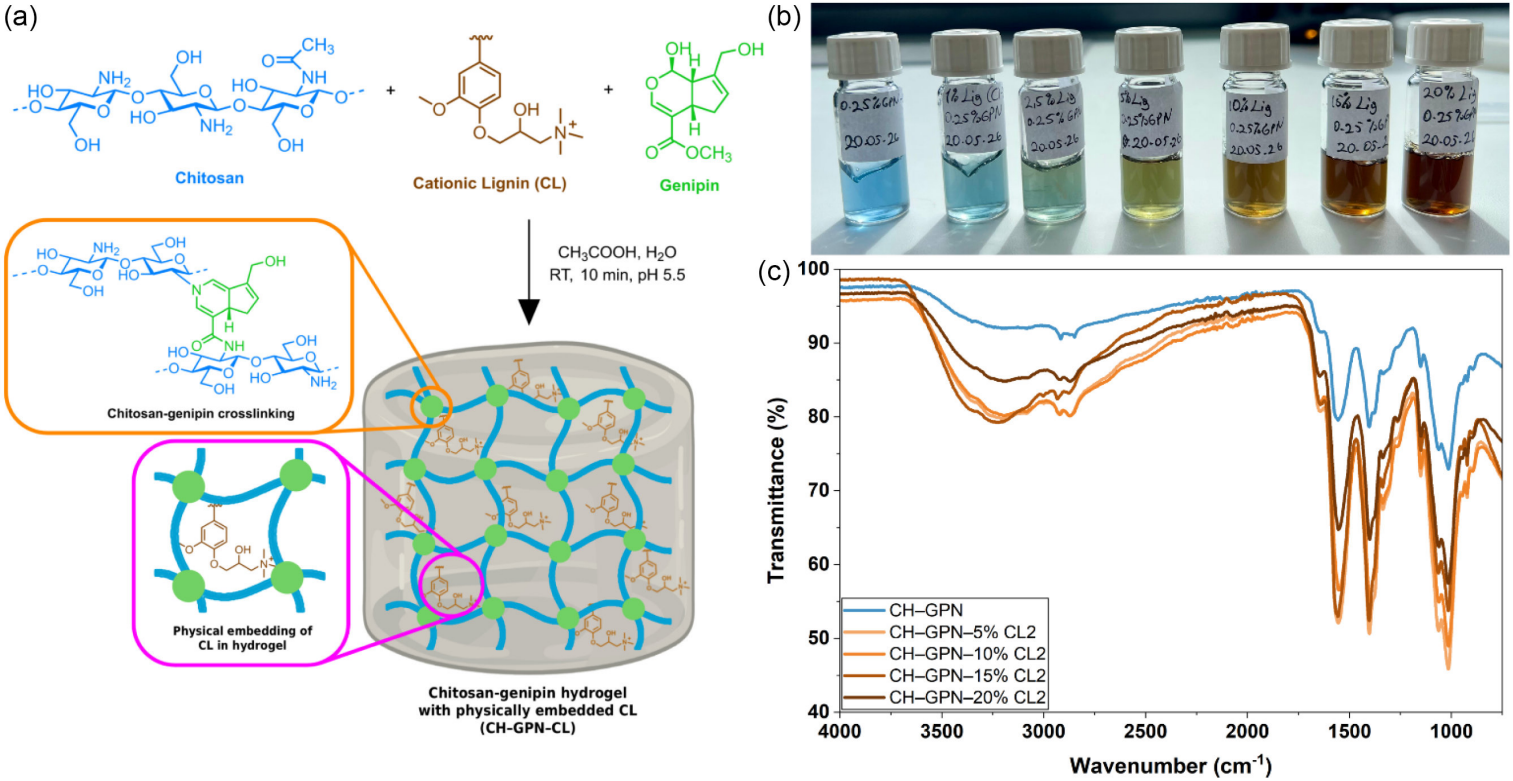
Preparation and visual appearance of CH–GPN–CL2 hydrogels. (a) Schematic representation of CH–GPN hydrogel formation and physical embedding of CL within the hydrogel network. (b) Photographs of a CH–GPN control hydrogel and CH–GPN–CL2 hydrogels prepared with varying CL2 contents. CH–GPN denotes the CH–GPN control hydrogel prepared at a GPN/CH ratio of 0.25% (w/w). (c) FTIR spectra of the CH–GPN control and CH–GPN–CL2 hydrogels with different CL2 contents.

The CL‐embedded hydrogels (CH–GPN–CL2) were then prepared with CL2 contents ranging from 1 to 20 wt% while keeping the GPN/CH ratio constant at 0.25%. The corresponding formulations are shown in Table S6. Gelation was observed for all CL2‐containing formulations, indicating that CL2 did not prevent network formation. Visually, increasing CL2 content resulted in a gradual color change from the blue CH–GPN control hydrogel to increasingly brown hydrogels (Figure 3b), consistent with the increasing lignin content. The characteristic blue color of the CH–GPN control hydrogel is consistent with previous reports on similar systems [24, 25].

FTIR analysis was used to compare the CH–GPN control and CH–GPN–CL2 hydrogels (Figure 3c). The spectra showed similar overall band shapes, although some differences in band intensity were observed, especially between the CH–GPN control and the CL2‐embedded hydrogels. These differences may be related to variations in sample morphology or drying state. Nevertheless, no distinct new bands were observed that would suggest additional covalent bonding between CL2 and the hydrogel matrix. This supports the interpretation that CL2 is mainly physically embedded within the CH–GPN network.

Overall, these results show that the CH–GPN system can accommodate CL2 over the tested concentration range and support its use as a matrix for physically embedding CL.

### 3.6 Hydrogel Characterization (Rheology and Morphology)

The viscoelastic properties of the CH‍–‍GPN control and CH–GPN–CL2 hydrogels were evaluated by frequency sweep rheology at 25 °C. The measurement at 25 °C was selected as a room‐temperature condition relevant to the intended plant application environment, while the corresponding measurements at 10 °C are shown in Figure S12. For all formulations, the storage modulus (*G*′) (Figure 4a) remained higher than the loss modulus (*G*″) (Figure 4b) across the tested frequency range, confirming predominantly elastic, gel‐like behavior. Compared to the CH–GPN control, the CL2‐containing hydrogels showed higher *G*′ values over most of the measured frequency range, indicating that CL2 incorporation influenced the mechanical properties of the hydrogel network. A similar trend was observed for *G*″, where CH–GPN–CL2 hydrogels generally showed higher viscous contributions than the control. This suggests that the physically embedded CL2 may increase the overall resistance of the network to deformation, without disrupting CH–GPN gel formation. However, CL2 incorporation did not lead to a simple concentration‐dependent increase in stiffness. Instead, the modulus values varied between formulations, indicating that the effect of CL2 on the measured hydrogel stiffness is not straightforward. The loss modulus increased with frequency for all samples, indicating a greater viscous contribution at higher deformation rates. Overall, the rheology results show that CL2 can be incorporated into CH–GPN hydrogels over the tested concentration range while maintaining gel‐like mechanical behavior. The higher moduli observed for the CL2‐containing hydrogels compared to the control further suggest that CL2 incorporation affects the hydrogel network without preventing gel formation. Furthermore, to assess time‐dependent rheological stability, additional frequency sweep measurements were performed after storing the hydrogels at 4 °C for 60 days and compared with measurements after 1 day (Figure S13). The 1‐ and 60‐day profiles showed only minor differences, and *G*′ remained higher than *G*″ for all formulations, indicating that the hydrogels retained predominantly elastic, gel‐like behavior after storage under the tested conditions.

**FIGURE 4:**
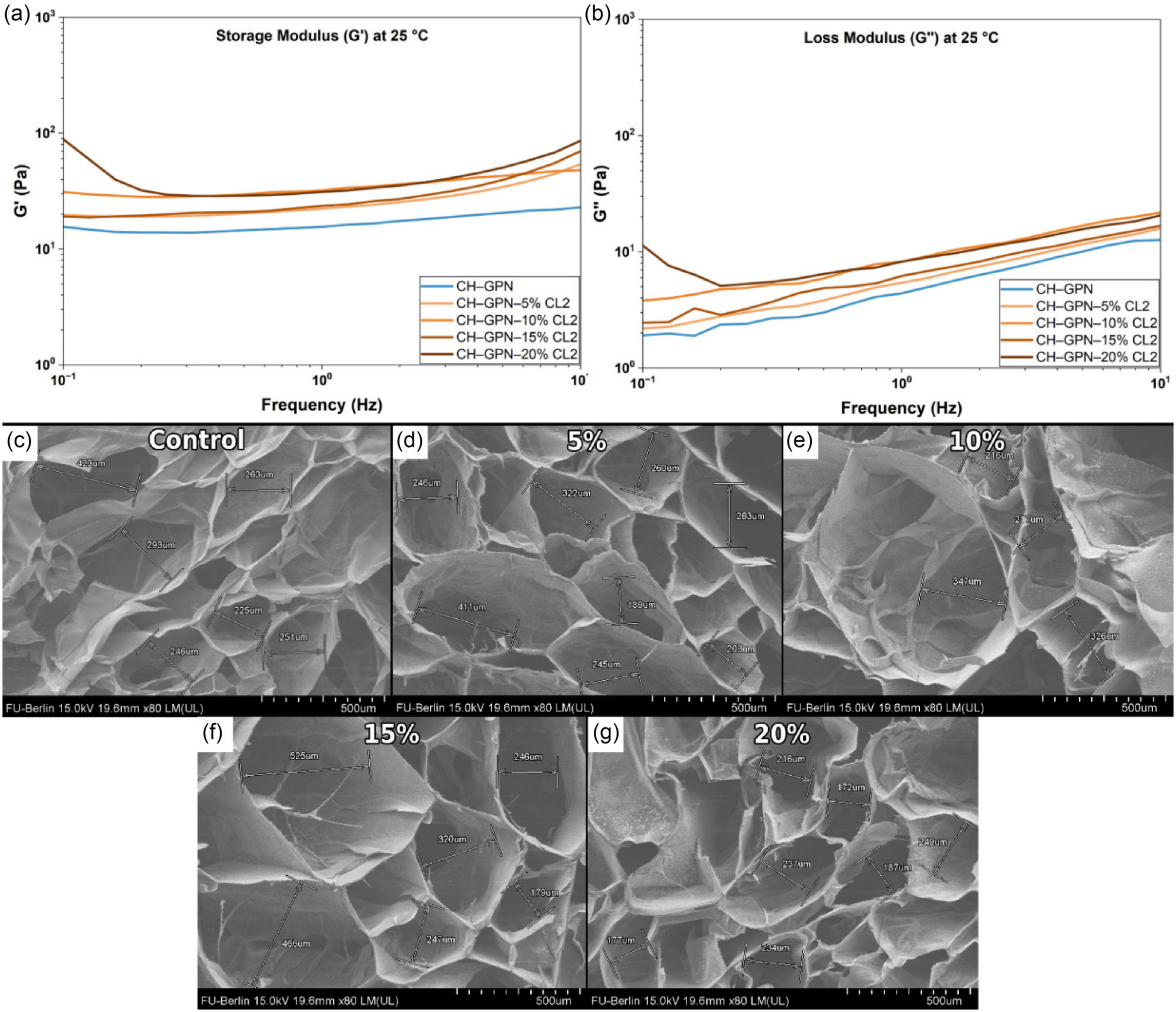
Rheological and morphological characterization of CH–GPN control and CH–GPN–CL2 hydrogels. Frequency sweep rheology at 25 °C showing (a) storage modulus (*G*′) and (b) loss modulus (*G*″) as a function of frequency for hydrogels containing different CL2 contents. SEM images of freeze‐dried hydrogels showing (c) CH–GPN control and CH–GPN–CL2 hydrogels containing (d) 5 wt%, (e) 10 wt%, (f) 15 wt%, and (g) 20 wt% CL2. Pore size measurements are indicated directly in the images.

The internal morphology of the hydrogels was further examined by SEM (Figure 4c–g). Both the CH–GPN control and CH–GPN‍–‍CL2 hydrogels showed a porous network structure after freeze‐drying. For the CL2‐containing hydrogels, porous morphologies were still observed across the tested formulations, with no obvious collapse of the freeze‐dried structure compared with the CH‍–‍GPN control. Some CL2‐containing samples appeared slightly more compact in selected regions; however, because SEM was performed on freeze‐dried samples, these differences should be interpreted only as qualitative morphological observations. Overall, the SEM results show that porous hydrogel morphologies were retained after CL2 incorporation under the tested preparation and imaging conditions.

### 3.7 CL2 Release and Swelling Capacity of CH–GPN–CL2 Hydrogels

Since CL2 was physically embedded within the CH–GPN hydrogel matrix, release experiments were performed to determine whether CL2 could become available in the surrounding aqueous medium. The release study was carried out in 10 mM MES + 10 mM MgCl_2_ buffer, matching the buffered conditions used for the plant infiltration assays. CL2 release was estimated by UV–vis spectroscopy at 280 nm, as lignin‐based compounds absorb in this region due to their aromatic structure [51, 52]. To ensure that the quantification was performed under matching buffer conditions, a CL2 standard curve was prepared in the same MES + MgCl_2_ release medium. The standard curve showed a linear response over the measured concentration range and was used to estimate CL2 concentrations in the release aliquots (Figure S14). The corresponding blank‐corrected absorbance values, estimated CL2 concentrations, and cumulative release values are shown in Table S7. Here, cumulative release refers to the total estimated amount of CL2 released up to a given time point, including both the CL2 remaining in the release medium and the CL2 removed during previous sampling steps. Because the UV–vis‐based values may be influenced by hydrogel‐derived absorbance and residual scattering, the release data are interpreted semiquantitatively and are used mainly to compare release trends across time points and CL2 loadings rather than as an absolute mass balance.

Using this cumulative calculation, the release profiles showed a rapid initial increase in the CL2‐associated absorbance signal, followed by a slower increase and stabilization after a few hours (Figure 5a). The 0.5 h time point was included to capture this early release phase, which likely corresponds to loosely associated or surface‐accessible CL2‐associated material diffusing from the hydrogel into the release medium. After this initial phase, the slower increase in cumulative release suggests a more gradual availability of CL2‐associated material from the swollen hydrogel matrix. The final estimated cumulative CL2 release after 5 h was also compared across the different CH–GPN–CL2 hydrogel formulations (Figure 5b), allowing the release signal to be evaluated as a function of CL2 content in the hydrogel.

**FIGURE 5:**
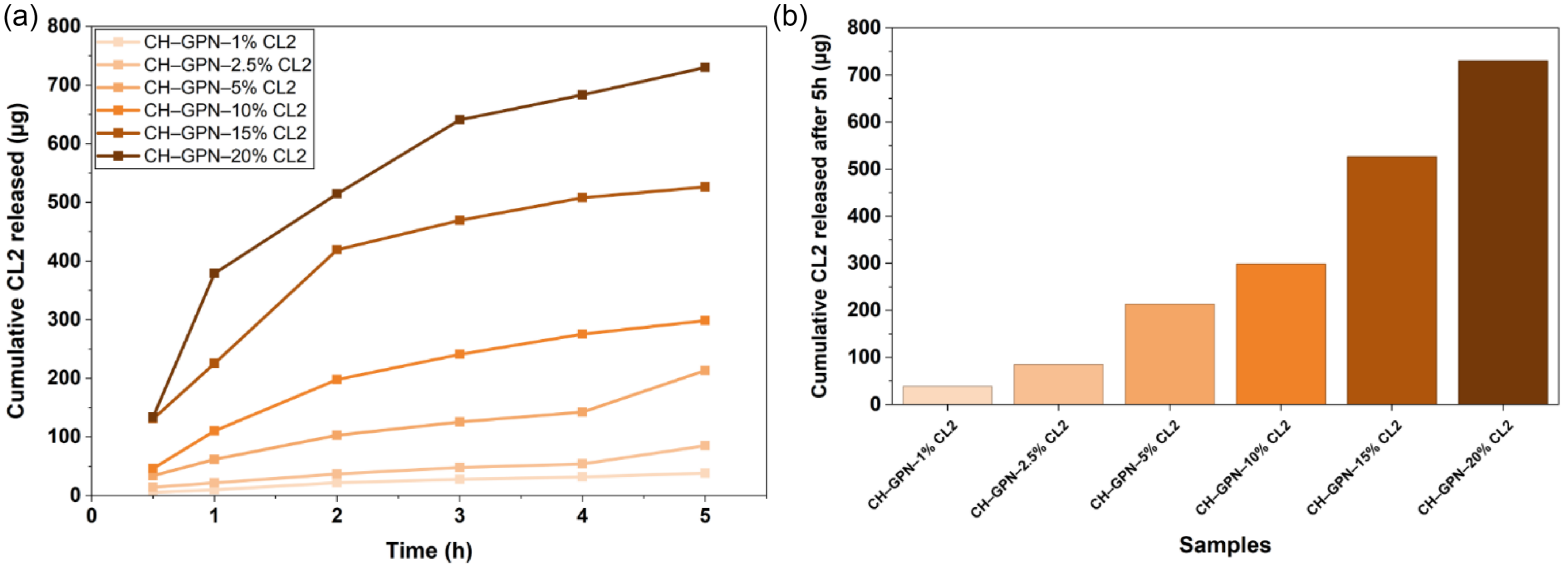
Estimated CL2 release from CH–GPN–CL2 hydrogels in 10 mM MES + 10 mM MgCl_2_ buffer. (a) Cumulative CL2 release from CH–GPN–CL2 hydrogels containing 1, 2.5, 5, 10, 15, and 20 wt% CL2 over 5 h, expressed as µg CL2 released per 250 mg wet hydrogel. (b) Final cumulative CL2 release after 5 h for each hydrogel formulation. CL2 concentrations were estimated from a CL2 standard curve prepared in the same release medium and measured at 280 nm. Data points represent the average of three repeated UV–vis measurements of the same aliquot from one release sample per formulation. The data are interpreted semiquantitatively due to possible contributions from hydrogel‐derived absorbance and residual scattering.

Preliminary longer‐term trials over several days showed substantial swelling and loss of defined macroscopic gel structure within the first day, which made reliable long‐term sampling and quantification difficult under these conditions. For this reason, the release experiment was focused on the first 5 h, where the hydrogel samples could still be handled more consistently. Nevertheless, the observed release shows that CL2 is not fully immobilized within the CH–GPN matrix and can become available in the surrounding medium, which is relevant for interpreting the antibacterial activity of the CL‐containing hydrogels.

Separately, water uptake and swelling capacity were evaluated using lyophilized CH–GPN and CH–GPN–CL2 hydrogels as an additional hydrogel characterization. These data were not directly combined with the CL2 release data, because swelling was measured using lyophilized hydrogel pieces in Milli‐Q water, whereas CL2 release was evaluated using wet hydrogels in MES + MgCl_2_ buffer. The swelling data therefore provide supporting information on water uptake and matrix swelling rather than direct release behavior. The hydrogels showed rapid water uptake within the first 10 min, followed by smaller changes up to 40 min, with swelling ratios ranging from approximately 15–30 for the included formulations (Figure S15 and Table S8).

### 3.8 Bacterial Inhibition Assay

The antimicrobial activity of CL and CH–GPN–CL2 hydrogels was evaluated against the plant pathogen *Pseudomonas syringae* pv. *tomato* DC3000 using CFU‐based survival assays, Live/Dead fluorescence staining, and SEM imaging (Figure 6). Initially, CL concentration‐dependent bacterial inhibition was tested over a concentration range of 5–500 µg mL^−1^, with bare lignin used as a control at the same concentrations. It was observed that GTMAC cationization significantly increased the antimicrobial activity of lignin by several orders of magnitude, particularly at higher concentrations (Figure 6a). This concentration‐dependent behavior is consistent with past work on *Escherichia coli* by Acurio Cerda et al. [14], where an increase in antibacterial activity was observed with increasing concentrations of CL, which was attributed to electrostatic interactions that damage the bacterial membranes. In addition, increasing the lignin/GTMAC ratio also increased bacterial inhibition, likely due to the higher surface charge and therefore stronger electrostatic interaction between the quaternary ammonium groups and the negatively charged bacterial membrane. At 100 µg mL^−1^, CL1 showed approximately 4‐log reduction, whereas CL2 and CL3 showed approximately 5‐log reduction. At 500 µg mL^−1^, the CFUs for CL3 were below the LOD. As CL2 showed strong antimicrobial activity while requiring a lower lignin/GTMAC mass ratio than CL3, it was selected for hydrogel development and further inhibition studies.

**FIGURE 6:**
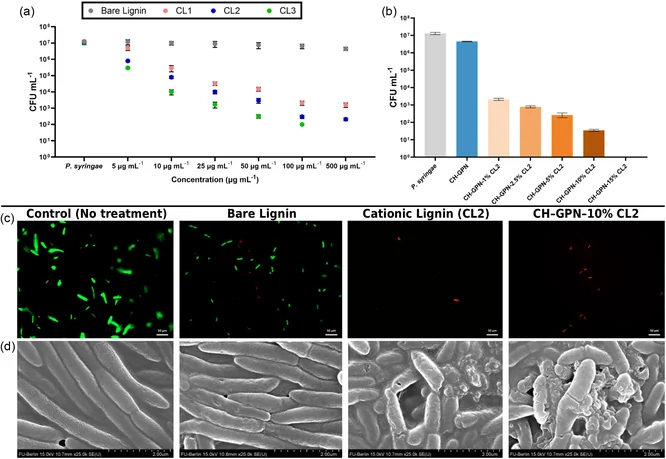
Antimicrobial activity and mechanistic evaluation against *Pseudomonas syringae* pv. *tomato* DC3000. (a) Concentration‐dependent bacterial inhibition by bare lignin and CL samples with different lignin/GTMAC ratios. (b) Bacterial inhibition by CH–GPN hydrogels containing different CL2 contents. (c) Live/Dead fluorescence microscopy images of *P. syringae* after treatment with (no treatment) control, bare lignin, CL2, and CH–GPN–10% CL2 hydrogel. Green fluorescence indicates cells with intact membranes, while red fluorescence indicates membrane‐compromised cells. (d) SEM images of *P. syringae* after the same treatments, showing changes in bacterial morphology and surface integrity. Scale bars: 10 µm in (c) and 2 µm in (d). Error bars in (a,b) represent ± SD from three independent biological replicates, each consisting of three technical replicates.

Figure 6b shows the antimicrobial activity of the CL‐embedded CH–GPN–CL2 hydrogels compared to mock‐treated *P. syringae* pv. *tomato* DC3000 and CH–GPN control hydrogel (0% CL2). The CH–GPN control exhibited some antimicrobial activity against *P. syringae*, which can be attributed to the known antimicrobial activity of genipin‐cross‐linked chitosan‐based hydrogels [25, 70]. Incorporation of CL2 into the hydrogel further increased bacterial inhibition, indicating that the antimicrobial function of CL2 is retained after physical embedding within the CH–GPN network. Hydrogels with 1% CL2 showed 4‐log microbial inhibition, while 10% CL2 resulted in 6‐log microbial inhibition. At 15% CL2, the recovered bacteria were below the LOD, with no bacterial colonies observed on the agar plate. At this point, it is also important to note that the 1% CL2 hydrogel contained 30 µg of CL, which produced around 3‐log inhibition in the free CL assay, as shown in Figure 6a. However, in its hydrogel form, a 4‐log inhibition was observed at a similar CL2 amount. This suggests that CL2 remains active even after incorporation into the hydrogel matrix. However, the present assay does not allow the contribution of CL2 to be fully separated from possible effects of the CH–GPN matrix or bacterial entrapment within the porous hydrogel network. Therefore, the improved inhibition is interpreted mainly as retained CL2 activity within the hydrogel rather than as evidence for a synergistic antimicrobial mechanism. The antibacterial activity of the hydrogels was also evaluated after two consecutive wetting/drying cycles. After cycling, the CH–GPN–CL2 hydrogels retained substantial CL2‐content‐dependent activity against *P. syringae* pv. *tomato* DC3000 (Figure S17). The 1%–10% CL2 formulations retained approximately 80%–85% of their initial antibacterial activity, while CH–GPN–15% CL2 maintained an approximately 5‐log reduction in viable bacteria. These results indicate that the hydrogels remain active after repeated wetting/drying under the tested laboratory conditions.

To further confirm our findings and gain insight into the antibacterial mechanism, Live/Dead fluorescence staining (Figure 6c) and SEM (Figure 6d) were performed following treatment of *P. syringae* with bare lignin at 100 µg mL^−1^, CL2 at 100 µg mL^−1^, and the CH–GPN–10% CL2. In Figure 6c, the untreated control displayed predominantly green fluorescence, indicating that most bacterial cells retained intact membranes. Consistent with this observation, SEM images in Figure 6d showed comparatively smooth, intact, rod‐shaped cells without obvious structural damage. Treatment with bare lignin resulted in only a modest increase in red fluorescence, while most cells remained green, suggesting limited membrane impairment. Similarly, SEM analysis of cells treated with bare lignin revealed only minor surface alterations, in agreement with the comparatively weak reduction in bacterial viability observed in the CFU assay.

In contrast, treatment with CL caused a pronounced reduction in green fluorescence and a corresponding increase in red‐stained cells, indicating substantial loss of membrane integrity. The associated SEM images revealed marked morphological alterations, including surface roughening and wrinkling, deformation of the bacterial envelope, and the presence of cell‐associated deposits and cellular debris. The most extensive effects were observed with cationic lignin hydrogels, with predominantly red fluorescence and very few viable green cells. SEM imaging further showed severely distorted bacterial morphologies, extensive surface disruption, irregular deposits, and abundant debris surrounding the cells. Together, the fluorescence and SEM observations corroborate the CFU results and demonstrate that cationic functionalization substantially enhances the antibacterial performance of lignin. The close correlation between morphological damage and reduced bacterial viability suggests that electrostatic interactions between the positively charged lignin‐based materials and the negatively charged bacterial cell surface disrupt the integrity of the cell envelope, ultimately resulting in membrane permeabilization and loss of bacterial viability.

The present antimicrobial assay was performed in vitro using *P. syringae* pv. *tomato* DC3000 as a representative plant pathogenic bacterial strain. The effect of the cationic lignin hydrogels on the beneficial plant‐associated bacterium *Sphingomonas melonis* Fr1 was also examined, with the corresponding results provided in the Supporting Information (Figure S16). Although increasing the CL2 content resulted in a concentration‐dependent reduction in viable bacterial counts, the inhibition was less pronounced (3‐log) than that observed against the phytopathogen *P. syringae* (6‐log), suggesting a degree of preferential antibacterial activity. This difference may arise from species‐specific differences in cell envelope properties, surface charge, and intrinsic stress tolerance, which could influence how each bacterium interacts with positively charged lignin‐based materials. However, further mechanistic studies would be required to determine why the inhibition was less pronounced for *S. melonis* Fr1. In addition, Acurio Cerda et al. reported that a related quaternary ammonium lignin showed no/minimal cytotoxicity toward human embryonic kidney cells (HEK293), with 90%–100% cell viability over the concentration range of 0–300 µg mL^−1^, while retaining strong antibacterial activity [14]. This concentration range overlaps with the free CL concentrations tested in the present study (5‍–‍500 µg mL^−1^). Future studies should evaluate the activity of CL and CH–GPN–CL2 hydrogels against a broader range of pathogens under in planta conditions and in more complex plant‐associated microbial systems, including possible effects on beneficial microorganisms, as well as cytotoxicity and safety‐relevant nontarget assays for the present formulation.

A comparison with commercial plant protection materials also requires application‐relevant disease models. For example, Meadows et al. reported that a grower standard based on copper/mancozeb alternated with streptomycin provided the most consistent control of bacterial diseases in tomato seedlings, including bacterial speck caused by *P. syringae* pv. *tomato*, whereas several tested biopesticides showed inconsistent or limited efficacy [71]. In addition, recent work on a carrier‐based single‐atom copper formulation showed that practical suitability was assessed not only by antimicrobial activity but also by comparison with commercial products such as Kocide 3000, Kasugamycin Copper, and zinc thiazole, together with phytotoxicity, copper release, soil residue, and plant disease‐control experiments [72]. In this context, the present CH–GPN–CL2 system should be considered an early‐stage bioactive formulation. Direct comparison with commercial plant protection materials will require side‐by‐side testing under in planta or leaf surface application conditions.

## 4 Conclusion

In this work, CL was synthesized by GTMAC cationization of kraft lignin and evaluated as a bio‐based antimicrobial material with potential relevance for plant protection applications. Successful cationization was confirmed by several analytical techniques, showing that increasing the lignin/GTMAC ratio generally increased nitrogen content, DS, molecular weight, and positive surface charge, although a plateau‐like saturation effect was observed at higher ratios. This suggests that further cationization becomes limited once the accessible reactive sites of lignin are increasingly consumed. Phytotoxicity assays using *Nicotiana benthamiana* showed that CL1–CL3 were well tolerated within the tested concentration range, with no clear concentration‐ or DS‐dependent phytotoxicity under buffered conditions. In bacterial inhibition assays against *P. syringae* pv. *tomato* DC3000, GTMAC cationization clearly improved the antimicrobial activity of lignin, with CL2 and CL3 showing strong inhibition. Based on its activity and lower lignin/GTMAC mass ratio compared to CL3, CL2 was selected for incorporation into CH–GPN hydrogels.

The CH–GPN network was able to physically embed CL2 without preventing gel formation. FTIR, rheology, and SEM results indicate that CL2 could be incorporated within the hydrogel matrix while preserving the overall gel‐like mechanical behavior and porous morphology. Release experiments further indicated that CL2‐associated material can become available from CH–GPN–CL2 hydrogels under aqueous buffer conditions, although these data should be interpreted semiquantitatively rather than as an absolute mass balance. The resulting CH–GPN–CL2 hydrogels showed increased in vitro antimicrobial activity compared to the CH–GPN control, while Live/Dead staining and SEM imaging further supported membrane damage as a likely antibacterial mechanism. Testing against the beneficial plant‐associated bacterium *Sphingomonas melonis* Fr1 showed less pronounced inhibition than against *P. syringae* pv. *tomato* DC3000. Overall, these results show that CL and CL‐embedded hydrogels are early‐stage bioactive materials for further evaluation in plant protection contexts. However, the present study is limited to material characterization, leaf infiltration compatibility assays, CL2 release testing under aqueous buffer conditions, and in vitro bacterial inhibition. Importantly, the present study does not establish a final application format for the hydrogel such as a sprayable coating, leaf‐adherent film, or injectable formulation. Future studies should evaluate the plant compatibility and performance of CL‐embedded hydrogels under in planta and application‐relevant conditions, including application to whole plants, leaf retention, release behavior, efficacy against pathogenic growth on plants, formulation stability, possible off‐target effects on beneficial microorganisms, and direct comparison with commercial plant protection products.

## Funding

This work was supported by the Volkswagen Foundation through project “Lignocide” (Project Number‐0071885).

## Conflicts of Interest

The authors declare no conflicts of interest.

## Supporting information

Supplementary Material

## Data Availability

The data that support the findings of this study are available from the corresponding author upon reasonable request.
