# Supplementary material for: Upcycling Industrial Lignin Into Cationic Hydrogels With Potential for Sustainable Plant Protection
Source: ChemSusChem. 2026 Oct 15;19(20):e71125. doi: 10.1002/cssc.71125 (PMC13671094; doi:10.1002/cssc.71125)
Supplement: Supplementary file 1 [file CSSC-19-e71125-s001.pdf]

SUPPORTING INFORMATION

Upcycling Industrial Lignin into Cationic Hydrogels with Potential for Sustainable Plant Protection

Darindra Sandya Thanos,[a] Zisan Caliskan Acik,[a] Emma Pauline Becker,[a] Max-Emanuel Zirngibl,[b] Tatyana L. Povolotsky,[a] Marcel Wiermer,[b] Mitja Remus-Emsermann,[b] Sanjam Chandna*[a]

[a] Institute of Chemistry and Biochemistry
Freie Universität Berlin
Arnimallee 22, 14195 Berlin, Germany

[b] Institute of Biology
Freie Universität Berlin
Königin-Luise-Straße 12-16, 14195 Berlin, Germany

* Email of corresponding author:
sanjam1994@zedat.fu-berlin.de

Table S1. Elemental analysis of bare lignin and cationic lignin (CL) samples, including reaction yield after purification and lyophilization. The degree of substitution (DS) was calculated from the N% content.

Sample	Lignin:GTMAC (w/w)	Yield (%)	N (%)	C (%)	H (%)	S (%)	DS (%)
Bare lignin	-	-	0.23	41.86	4.682	1.343	-
CL1	1:1	59.39	2.41	50.11	5.686	0.847	41.92
CL2	1:2	59.61	2.60	48.91	5.835	0.723	46.52
CL3	1:3	59.46	3.00	57.92	6.758	0.862	57.11
CL4	1:4	66.53	3.16	57.66	6.883	0.908	61.74
CL5	1:5	61.68	3.06	53.36	6.376	0.727	58.82

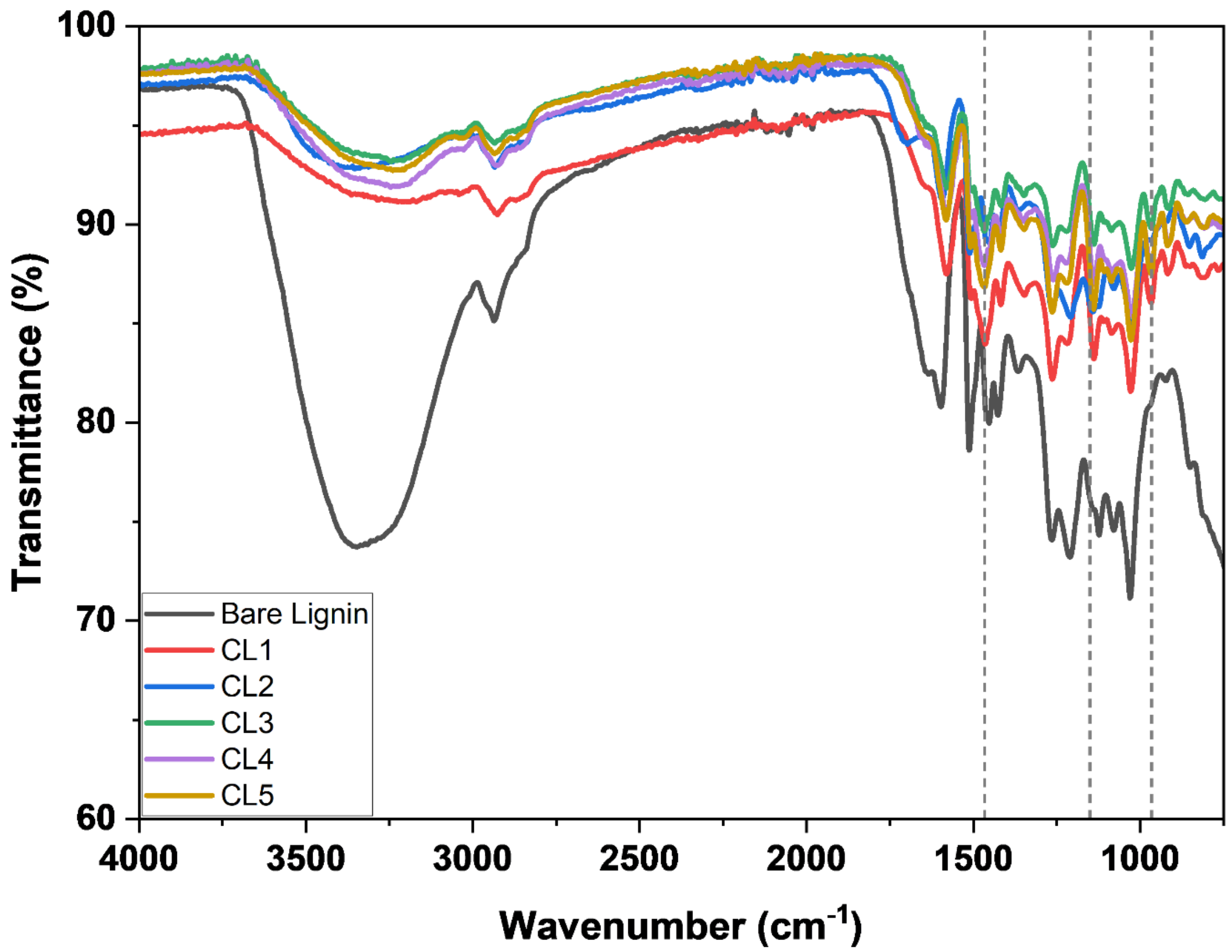

Figure S1. Full FTIR spectra (4000-750 cm^{-1}) of bare lignin and cationic lignin (CL) samples. Dashed lines highlight key bands associated with GTMAC modification (Grey dashed lines at 1466, 1150, and 966 cm^{-1}).

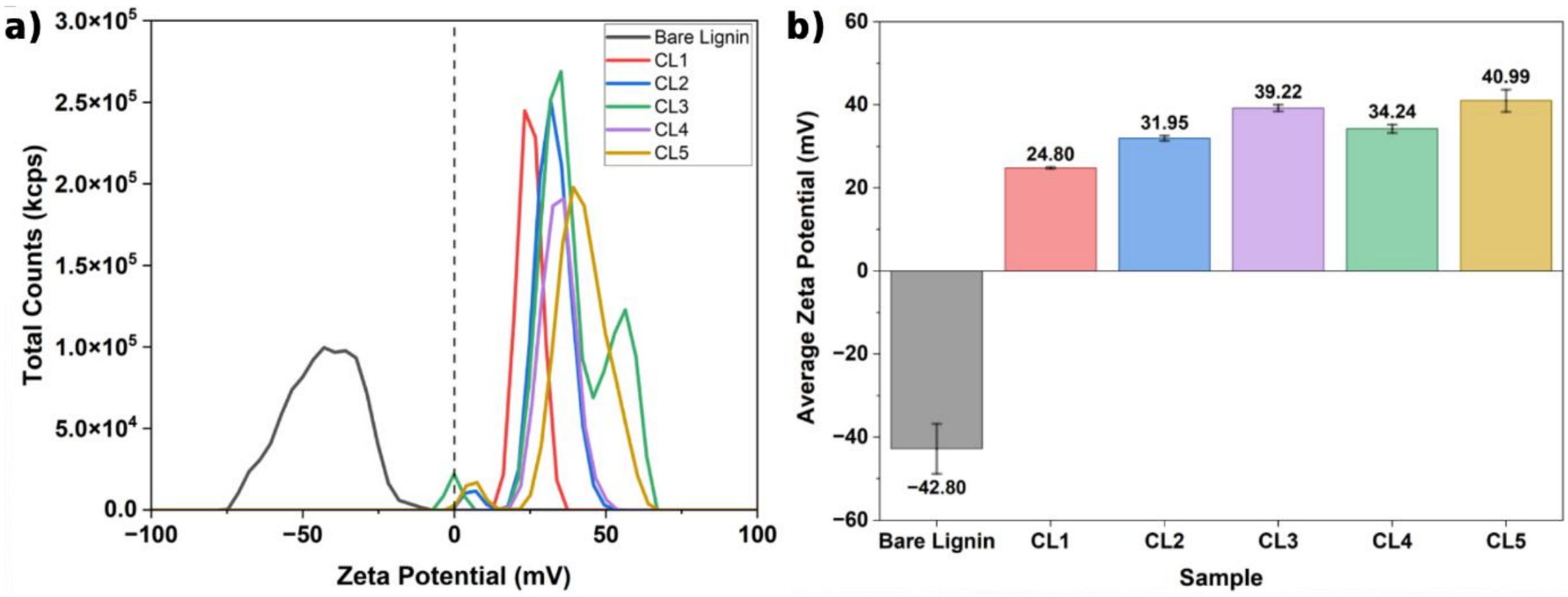

Figure S2. Zeta potential analysis of bare lignin and cationic lignin (CL) samples. a) Zeta potential distributions shown as count rate versus zeta potential. b) Average zeta potential values of bare lignin and CL samples. Error bars indicate standard deviation from three independent replicate solutions (n = 3).

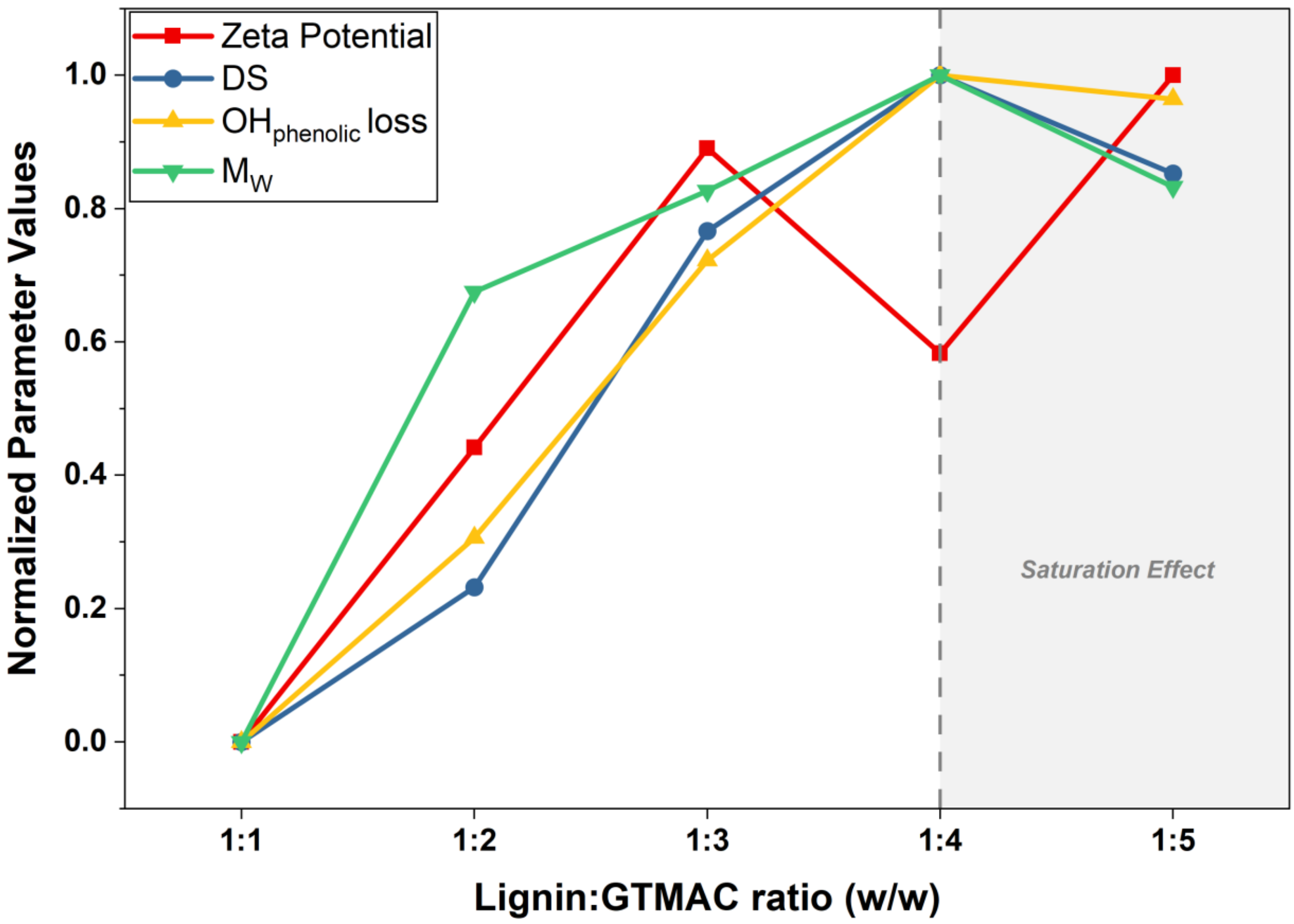

Figure S3. Normalized comparison of zeta potential, degree of substitution (DS), molecular weight (M_w), and relative phenolic hydroxyl loss for CL1–CL5 as a function of lignin:GTMAC mass ratio. The plateau-like behavior observed at ratios 1:4 and 1:5 suggests saturation of accessible reactive hydroxyl groups during lignin cationization.

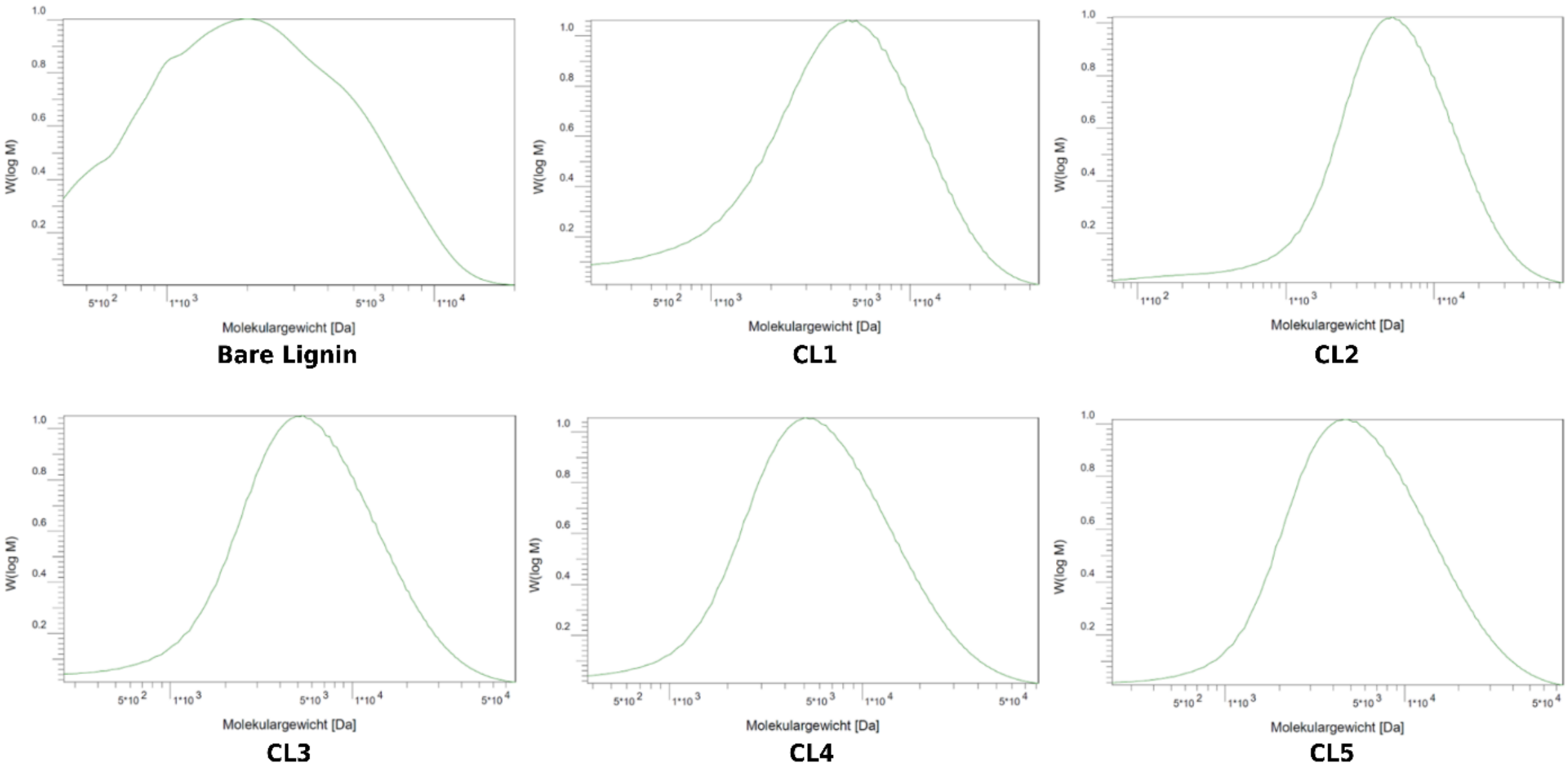

Figure S4. Gel permeation chromatography (GPC) trace of bare lignin and CL samples, showing the molecular weight distribution as W(log M) versus molecular weight (Da). Measurements were performed in DMSO containing 0.075 M $NaNO_3$ using refractive index detection.

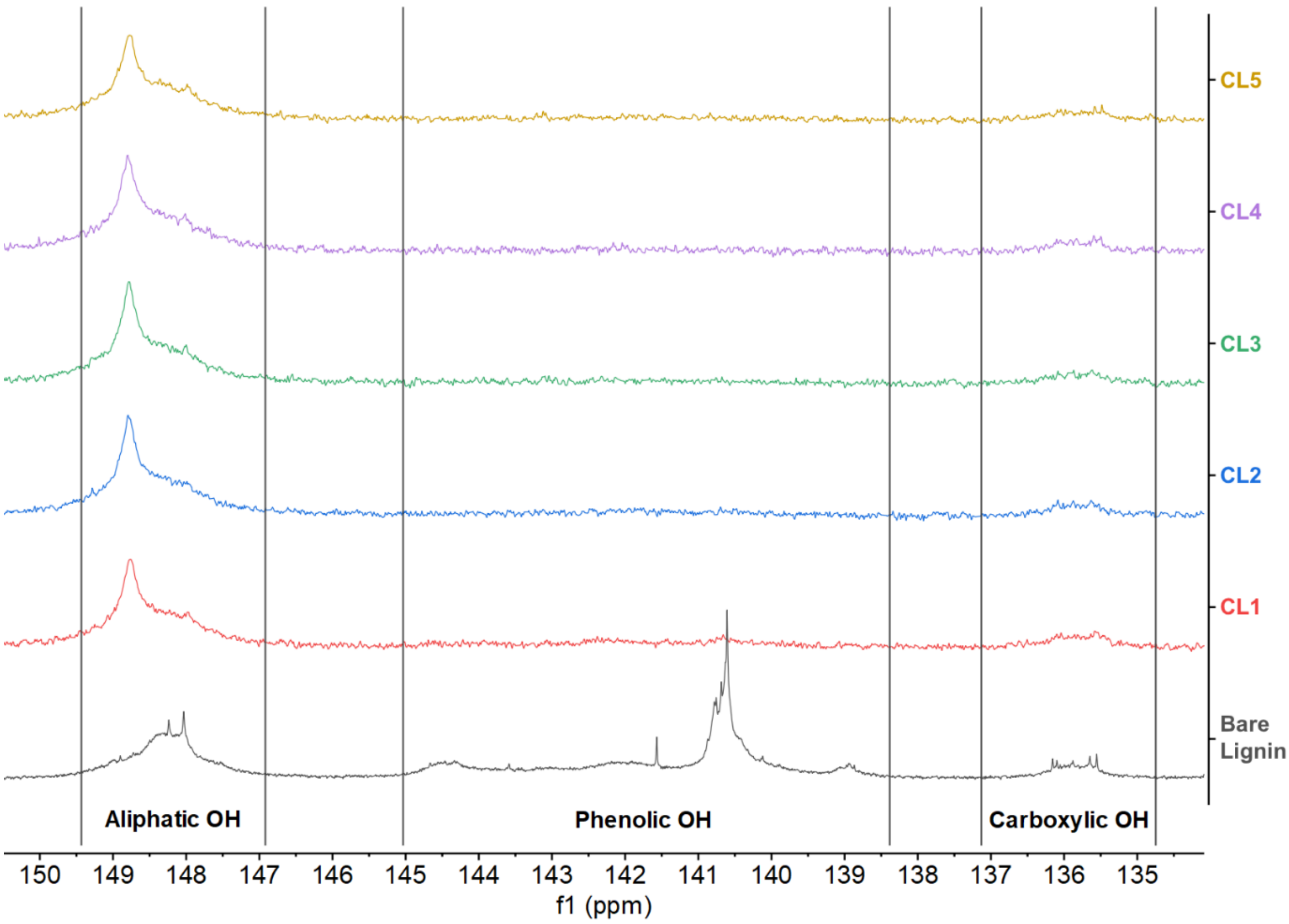

Figure S5. Stacked ^{31}P NMR spectra of bare lignin and cationic lignin samples (CL1–CL5) after phosphitylation with TMDP. Vertical reference lines indicate the characteristic chemical shift regions corresponding to aliphatic, phenolic, and carboxylic hydroxyl groups. The reduced phenolic OH signal intensity observed for the CL samples compared to bare lignin is consistent with hydroxyl group consumption during lignin cationization.

Table S2. Quantified phenolic and aliphatic hydroxyl group concentrations of bare lignin and all CL samples determined by ^{31}P NMR spectroscopy after phosphitylation with TMDP.

Sample	Lignin:GTMAC (w/w)	Phenolic OH (mmol g^{-1})	Aliphatic OH (mmol g^{-1})
**Bare lignin**	-	3.95	2.32
**CL1**	1:1	1.00	3.03
**CL2**	1:2	0.57	3.13
**CL3**	1:3	0.37	2.94
**CL4**	1:4	0.29	2.52
**CL5**	1:5	0.44	3.19

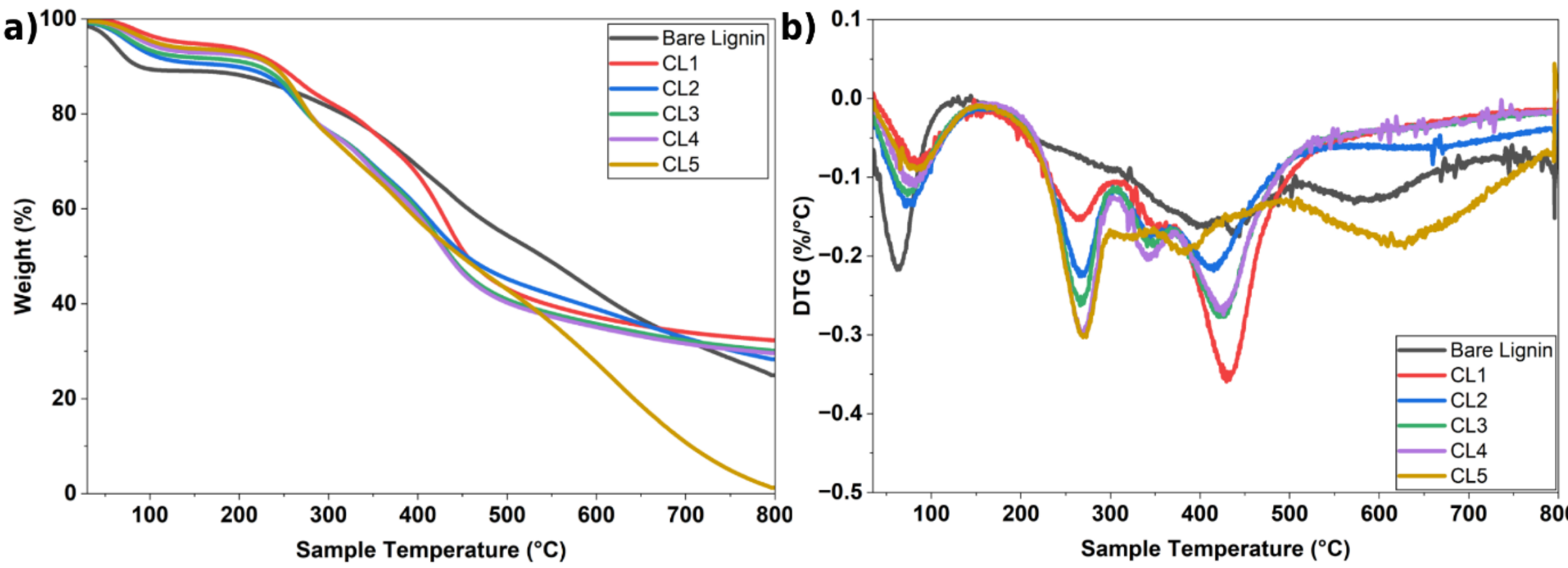

Figure S6. Thermogravimetric analysis (TGA) of bare lignin and cationic lignin samples (CL1–CL5). **a)** TGA curves showing weight loss (%) as a function of temperature (°C). **b)** Derivative thermogravimetric (DTG) curves showing the rate of weight loss (% °C^{-1}) as a function of temperature (°C).

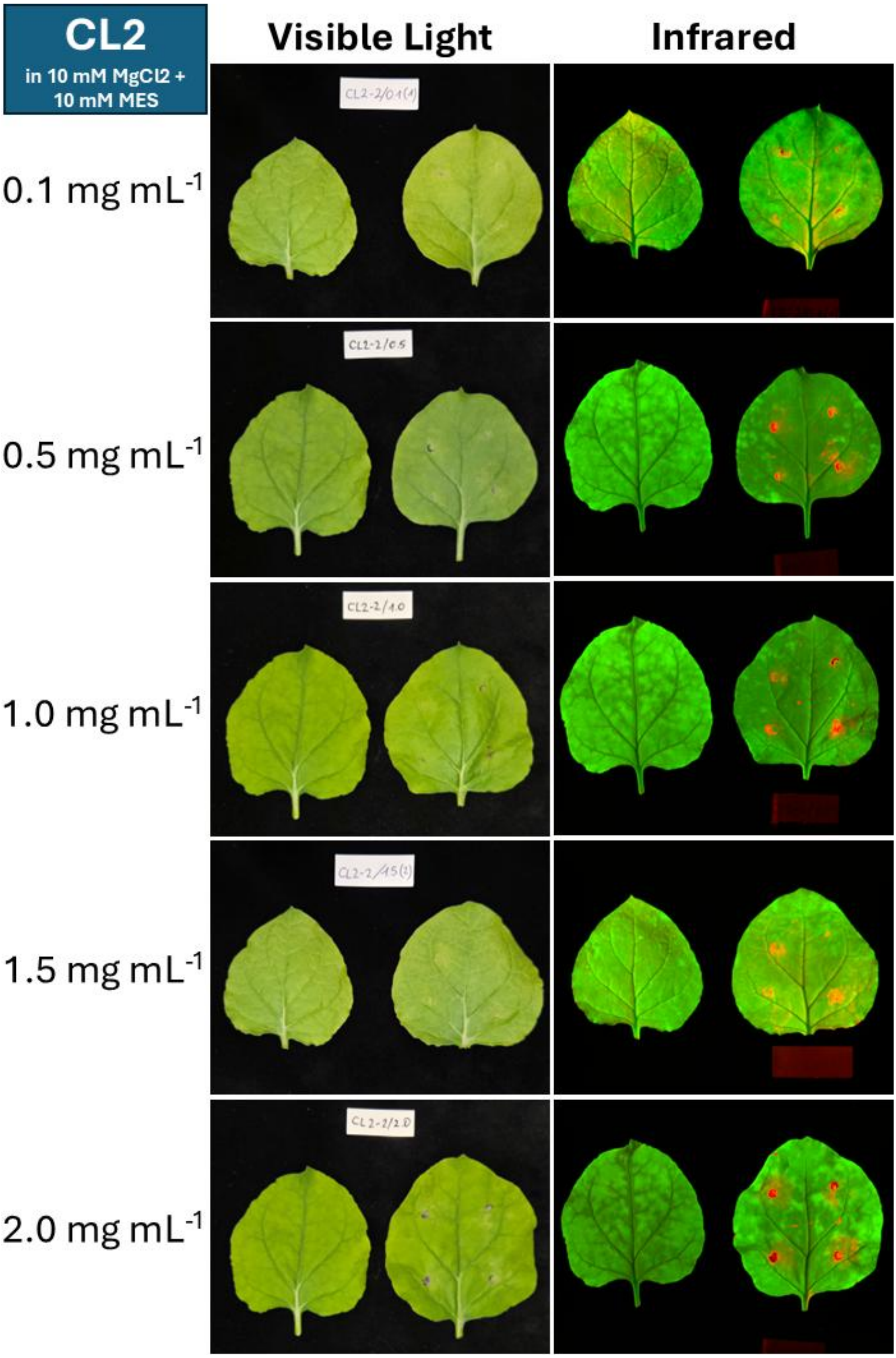

Figure S7. Leaf infiltration-based phytotoxicity assay of *Nicotiana benthamiana* leaves treated with CL2 at varying concentrations (0.1–2.0 mg mL^{-1}), dissolved in 10 mM $MgCl_2$ + 10 mM MES. The same buffer was used as the control infiltration spot (top right position on each treated leaf). For each picture, the left leaf represents the untreated control, while the right leaf shows the treated sample. Within each concentration, images are presented as visible light (left) and its corresponding infrared image (right).

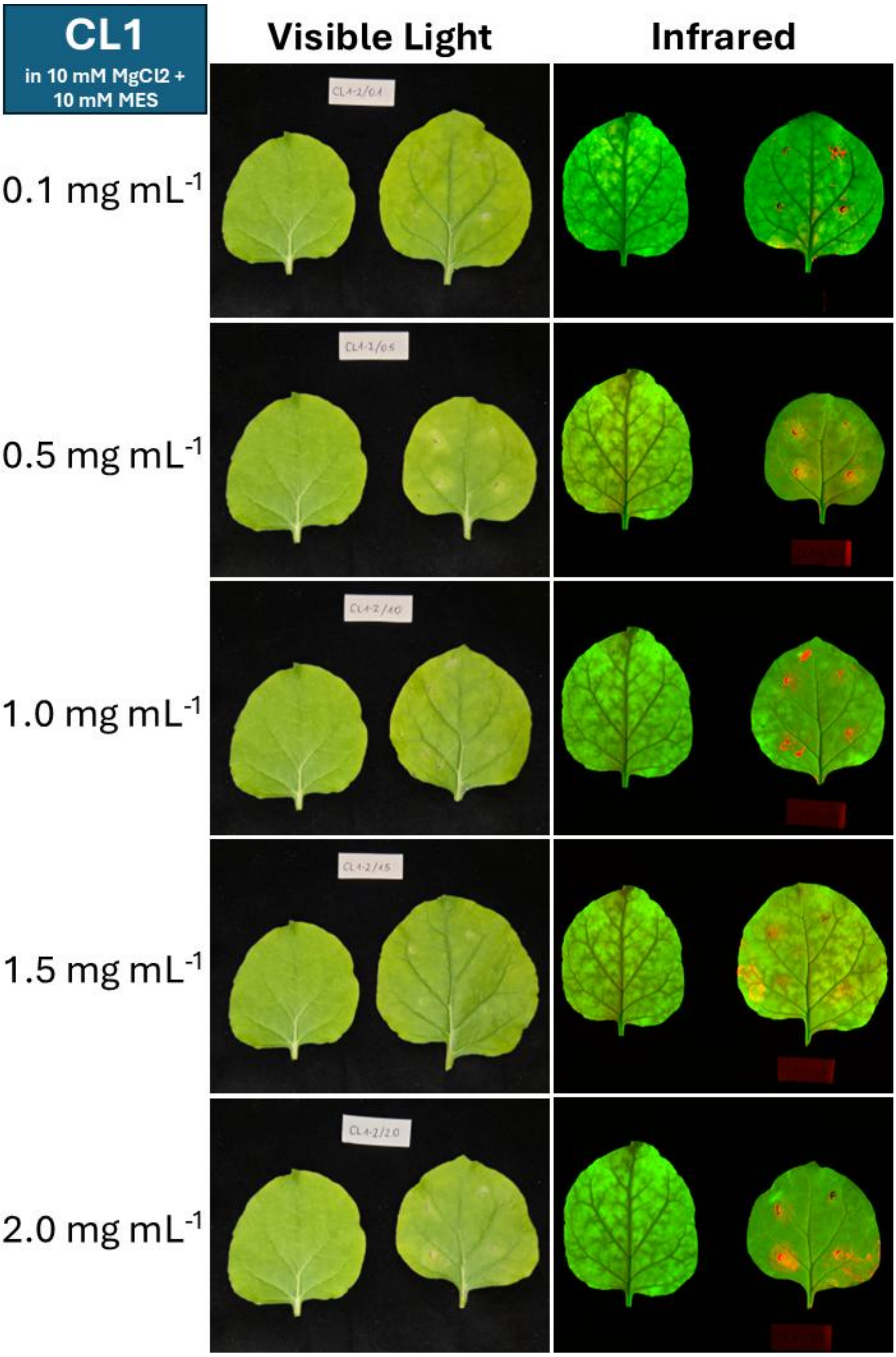

Figure S8. Leaf infiltration-based phytotoxicity assay of *Nicotiana benthamiana* leaves treated with CL1 at varying concentrations (0.1–2.0 mg mL^{-1}), dissolved in 10 mM $MgCl_2$ + 10 mM MES. The same buffer was used as the control infiltration spot (top right position on each treated leaf). For each picture, the left leaf represents the untreated control, while the right leaf shows the treated sample. Within each concentration, images are presented as visible light (left) and its corresponding infrared image (right).

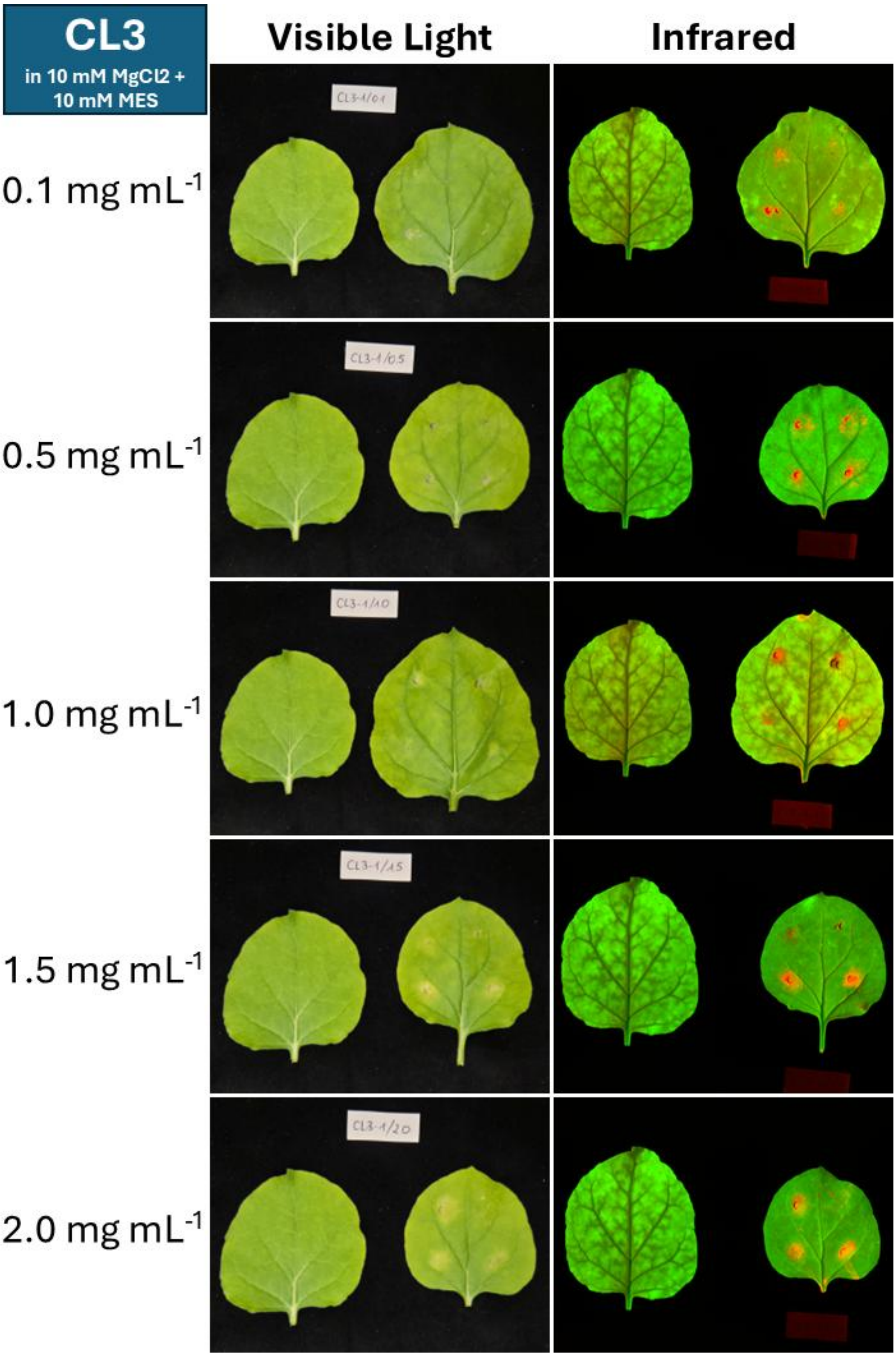

Figure S9. Leaf infiltration-based phytotoxicity assay of *Nicotiana benthamiana* leaves treated with CL3 at varying concentrations (0.1–2.0 mg mL^{-1}), dissolved in 10 mM $MgCl_2$ + 10 mM MES. The same buffer was used as the control infiltration spot (top right position on each treated leaf). For each picture, the left leaf represents the untreated control, while the right leaf shows the treated sample. Within each concentration, images are presented as visible light (left) and its corresponding infrared image (right).

Table S3. Electrolyte leakage data from *Nicotiana benthamiana* leaf discs after infiltration with CL samples. Conductivity values were recorded for CL1, CL2, CL3, and the 10 mM $MgCl_2$ + 10 mM MES mock control after 5 days of infiltration. For each biological replicate, conductivity was measured immediately after transfer into fresh water (0 h), after 4 h of incubation, and after heat treatment to determine the maximum conductivity (C_{max}). Electrolyte leakage was calculated according to Equation 3 in the main text. Mean electrolyte leakage values and standard deviations were calculated from three independent biological replicates.

Sample	Concentration (mg mL^{-1})	Biological Replicate 1				Biological Replicate 2				Biological Replicate 3			
		Conductivity (μS cm^{-1})			Electrolyte Leakage (%)	Conductivity (μS cm^{-1})			Electrolyte Leakage (%)	Conductivity (μS cm^{-1})			Electrolyte Leakage (%)
		0 h	4 h	Max.		0 h	4 h	Max.		0 h	4 h	Max.	
Control	-	3	3	22	13.64	0	2	24	8.33	0	4	27	14.81
CL1	0.1	4	6	25	24.00	1	3	27	11.11	0	3	27	11.11
	0.5	3	4	29	13.79	0	1	23	4.35	0	3	27	11.11
	1.0	3	7	33	21.21	0	3	25	12.00	0	3	23	13.04
	1.5	3	5	29	17.24	0	2	22	9.09	0	2	24	8.33
	2.0	3	4	28	14.29	0	2	33	6.06	0	3	29	10.34
CL2	0.1	3	5	32	15.63	0	2	24	8.33	1	4	23	17.39
	0.5	3	4	31	12.90	0	2	30	6.67	1	3	24	12.50
	1.0	3	3	29	10.34	0	2	28	7.14	0	2	20	10.00
	1.5	3	3	24	12.50	0	1	21	4.76	0	2	22	9.09
	2.0	3	4	24	16.67	0	4	26	15.38	0	4	25	16.00
CL3	0.1	3	4	24	16.67	0	4	33	12.12	1	6	30	20.00
	0.5	3	3	24	12.50	0	2	32	6.25	0	2	26	7.69
	1.0	3	3	25	12.00	0	2	27	7.41	0	4	22	18.18
	1.5	3	3	25	12.00	0	2	26	7.69	0	2	22	9.09
	2.0	4	6	26	23.08	0	3	26	11.54	1	4	27	14.81

Table S4. One-way ANOVA of electrolyte leakage data from Nicotiana benthamiana leaf discs after CL infiltration. Electrolyte leakage values (%) from the $MgCl_2$/MES buffer control and all CL-treated groups in Table S3 were analyzed using one-way ANOVA. The analysis included three independent biological replicates per group (n = 3).

Source	DF	Sum of Squares	Mean square	F value	P value
**Model**	15	372.69613	24.84641	1.29354	0.26204
**Error**	32	614.65767	19.20805	—	—
**Total**	47	987.35379	—	—	—

One-way ANOVA showed no significant differences among the $MgCl_2$ + MES control and CL-treated groups (p = 0.262). Thus, no post-hoc multiple-comparison test was applied.

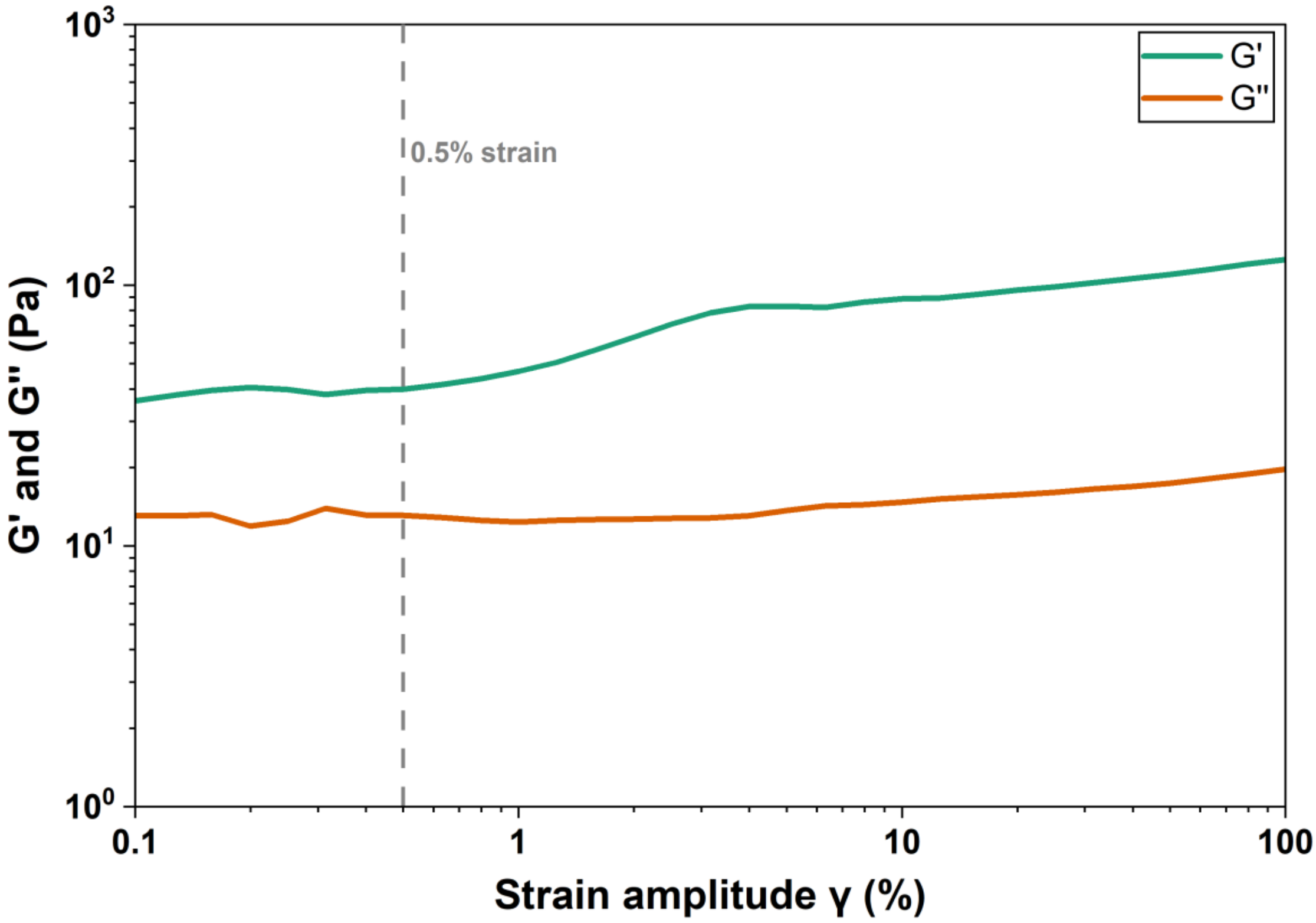

Figure S10. Strain sweep measurement of a representative hydrogel sample showing the storage modulus (G′) and loss modulus (G″) as a function of strain amplitude. Frequency sweep measurements were subsequently conducted at a constant strain of 0.5% within the low-strain viscoelastic region.

Table S5. Optimization of chitosan-genipin hydrogel formulations with varying genipin content. All formulations contained 6 mL of chitosan solution (1.5% w/v in 1% v/v acetic acid) and were adjusted to a final volume of 7 mL using Milli-Q water. Genipin was added from an aqueous stock solution (0.1% w/v), and the GPN/CH value represents the mass ratio of genipin to chitosan. Grey-shaded rows indicate formulations that did not result in gelation after curing.

Sample	CH solution (mL)	GPN solution (mL)	Milli-Q water (mL)	GPN/CH (w/w, %)	Gelation
**CH–GPN0.05**	6	0.045	0.955	**0.05**	No
**CH–GPN0.10**	6	0.090	0.910	**0.10**	No
**CH–GPN0.15**	6	0.135	0.865	**0.15**	Yes
**CH–GPN0.20**	6	0.180	0.820	**0.20**	Yes
**CH–GPN0.25**	6	0.225	0.775	**0.25**	Yes
**CH–GPN0.50**	6	0.450	0.550	**0.50**	Yes
**CH–GPN1.00**	6	0.900	0.100	**1.00**	Yes

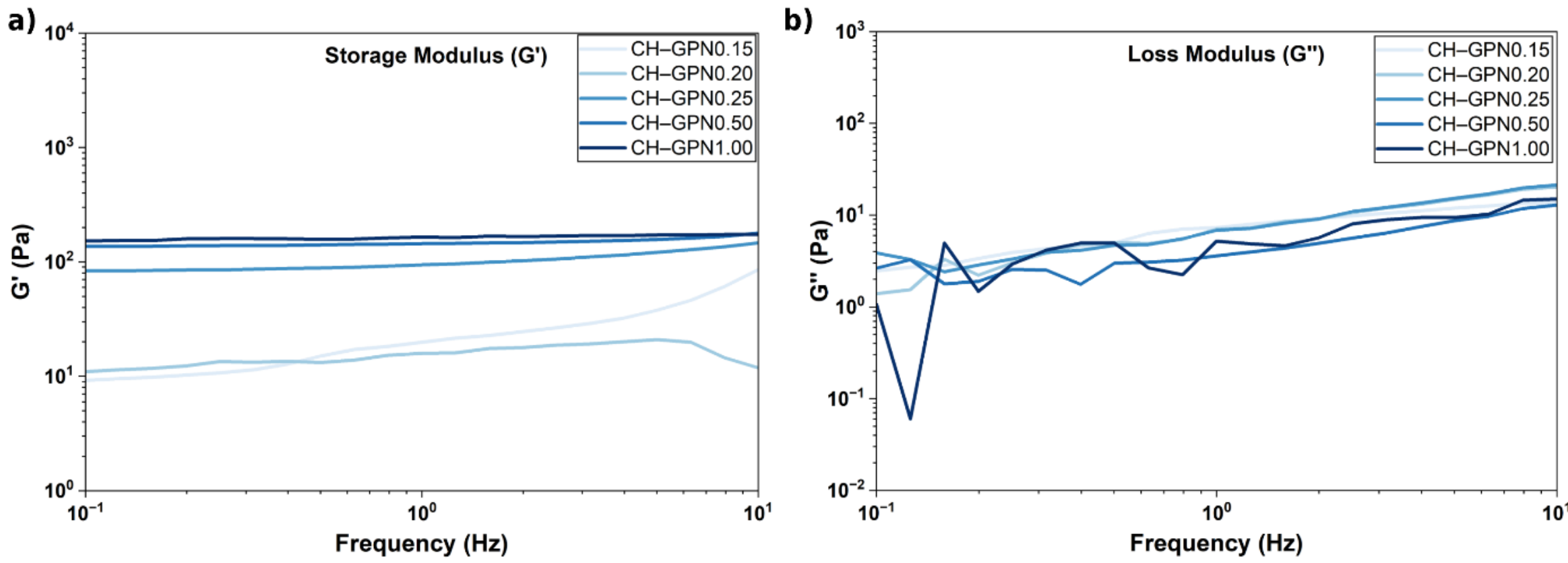

Figure S11. Rheological analysis of chitosan-genipin (CH–GPN) hydrogel formulations prepared with varying genipin contents. Frequency sweep measurements were performed at 10 °C for formulations that formed self-supporting gels during optimization. **a)** Storage modulus (G′) and **b)** loss modulus (G″) as a function of frequency. Sample labels indicate the GPN/CH mass ratio expressed in % (w/w).

Table S6. Formulations of CH–GPN–CL2 hydrogels with varying CL2 contents. All formulations were prepared using 6 mL of chitosan solution (1.5% w/v in 1% v/v acetic acid) and genipin from an aqueous stock solution (0.1% w/v), with the GPN/CH ratio kept constant at 0.25% (w/w). CL2 content was varied from 1 to 20 wt%, calculated relative to the chitosan mass, by adjusting the volumes of 20 mg mL^{-1} aqueous CL2 stock solution and Milli-Q water. The GPN/CH value represents the genipin-to-chitosan mass ratio.

Sample	CH solution (mL)	GPN solution (mL)	Milli-Q water (mL)	CL2 solution (mL)	GPN/CH (w/w, %)	CL2 (w/w, %)
**CH–GPN**	6	0.225	0.900	0.000	**0.25**	**-**
**CH–GPN–1% CL2**	6	0.225	0.855	0.045	**0.25**	**1**
**CH–GPN–2.5% CL2**	6	0.225	0.788	0.112	**0.25**	**2.5**
**CH–GPN–5% CL2**	6	0.225	0.675	0.225	**0.25**	**5**
**CH–GPN–10% CL2**	6	0.225	0.450	0.450	**0.25**	**10**
**CH–GPN–15% CL2**	6	0.225	0.225	0.675	**0.25**	**15**
**CH–GPN–20% CL2**	6	0.225	0.000	0.900	**0.25**	**20**

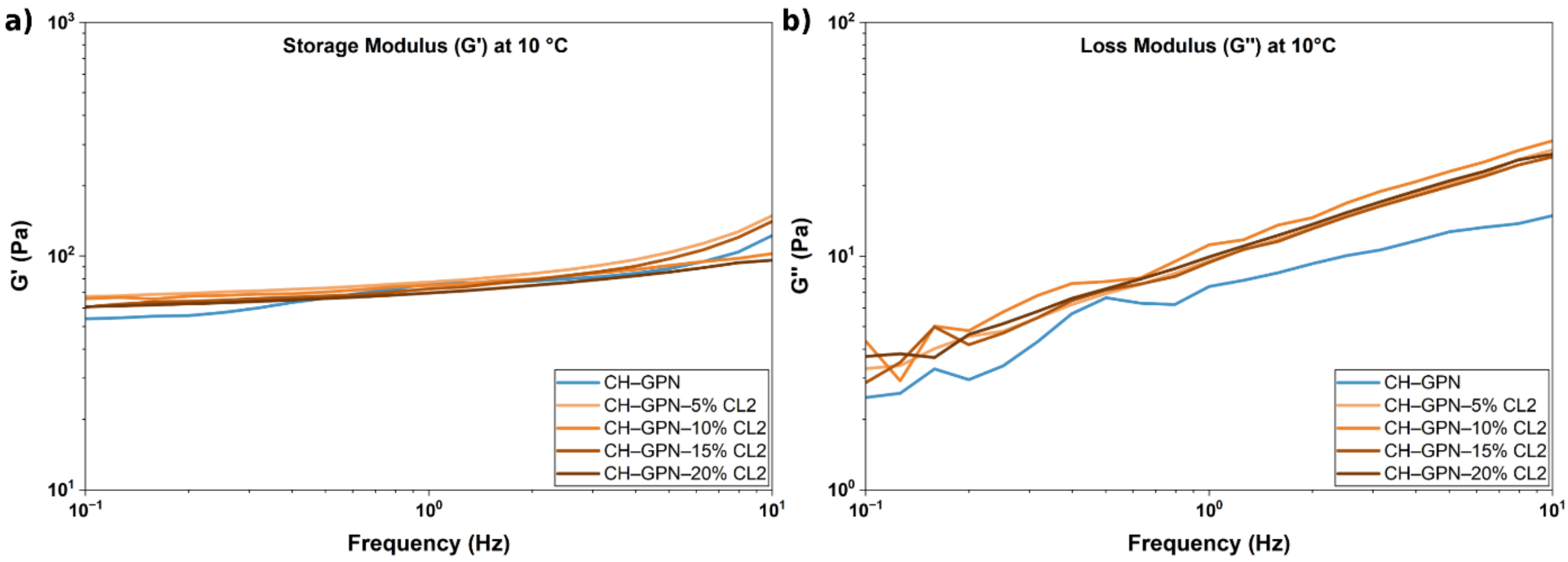

Figure S12. Frequency sweep rheology of CH–GPN control and CH–GPN–CL2 hydrogels at 10 °C. **a)** Storage modulus (G′) and **b)** loss modulus (G″) as a function of frequency for hydrogels containing

different CL2 loadings. For all formulations, G′ remained higher than G″ across the measured frequency range, indicating predominantly elastic, gel-like behavior under cooler conditions.

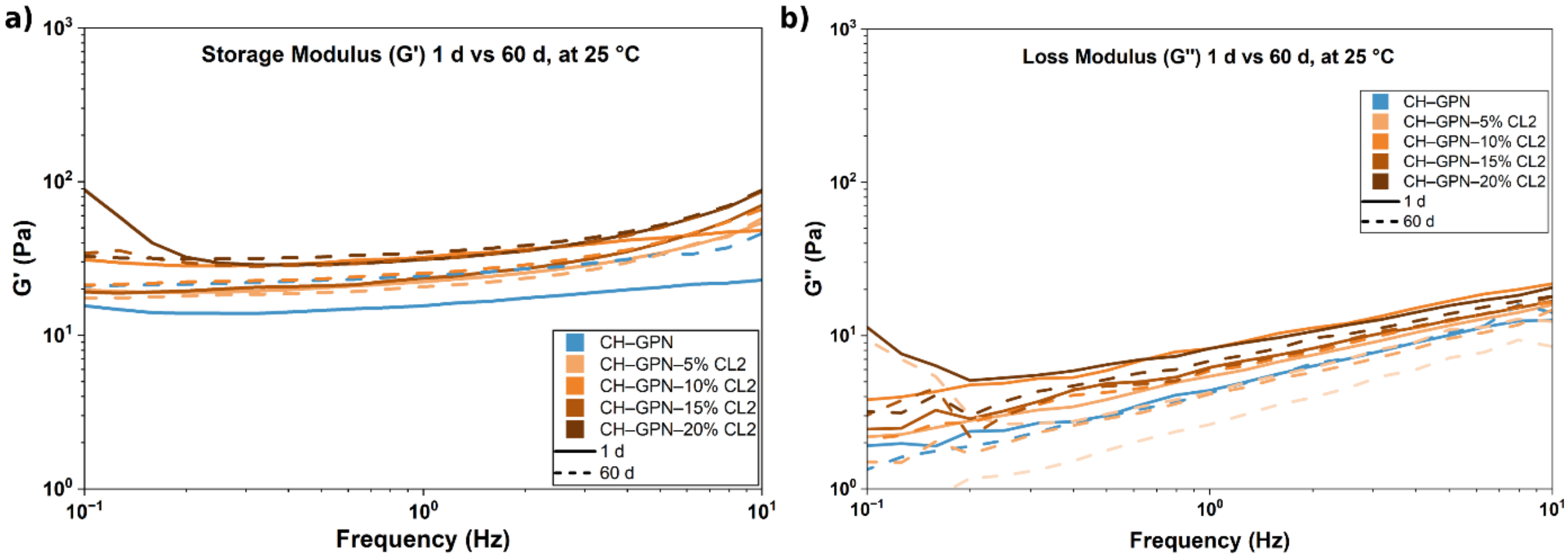

Figure S13. Time-dependent rheological comparison of CH–GPN control and CH–GPN–CL2 hydrogels after 1 day and 60 days of storage at 4 °C. Frequency sweep measurements were performed at 25 °C for hydrogels containing different CL2 loadings. **a)** Storage modulus (G′) and **b)** loss modulus (G″) as a function of frequency. Solid lines represent measurements after 1 day, while dashed lines represent measurements after 60 days. Across all formulations and both time points, G′ remained higher than G″ over the measured frequency range, indicating that the hydrogels retained predominantly elastic, gel-like behavior after 60 days under the tested conditions.

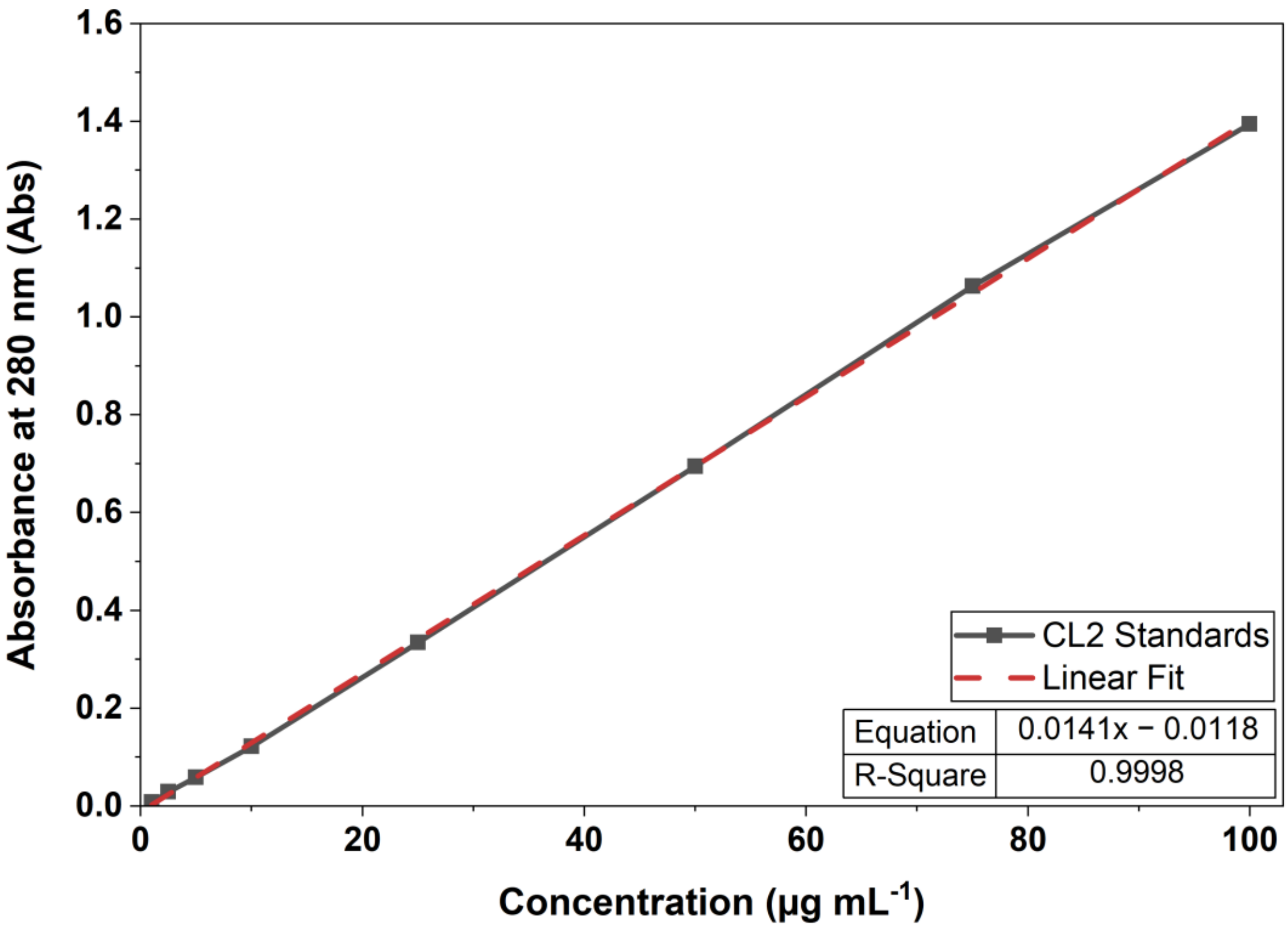

Figure S14. CL2 standard curve prepared in 10 mM MES + 10 mM $MgCl_2$ buffer using CL2 concentrations of 1, 2.5, 5, 10, 25, 50, 75, and 100 μg mL^{-1}. Absorbance was measured at 280 nm for UV–Vis-based estimation of CL2 release. The linear fit, corresponding equation, and R^2 value are shown in the graph, and the resulting calibration equation was used to estimate CL2 concentrations in release aliquots collected from CH–GPN–CL2 hydrogels.

Equation S1. Calculation of cumulative CL2 release during the release experiment with medium replacement. The cumulative amount of CL2 released at time point *n* was calculated as:

$$Q_n = C_n V_0 + V_s \sum_{i=1}^{n-1} C_i$$

where Q_n is the cumulative amount of CL2 released at time point *n* (µg), C_n is the CL2 concentration measured in the release medium at time point *n* (µg mL^{-1}), V_0 is the total volume of release medium (10 mL), V_s is the sampled and replaced volume at each time point (2 mL), and C_i represents the CL2 concentration in previously removed aliquots (µg mL^{-1}). This correction accounts for CL2 removed during earlier sampling steps.

Table S7. UV–Vis data used to estimate CL2 release from CH–GPN–CL2 hydrogels in 10 mM MES + 10 mM $MgCl_2$ buffer. Blank-corrected A_{280} values were obtained by subtracting the absorbance of CH–GPN hydrogel supernatants from the corresponding CH–GPN–CL2 release aliquots. CL2 concentrations were estimated using the calibration equation in **Figure S14**, and cumulative release was calculated using Equation S1 to account for medium replacement. Values are reported as CL2 release per 250 mg wet hydrogel and were calculated from the average of three repeated UV–Vis measurements of each aliquot. Data are interpreted semi-quantitatively due to possible contributions from hydrogel-derived absorbance and residual scattering.

Hydrogel formulation	Time (h)	Blank-corrected A_{280} (Abs)	Estimated CL2 concentration (µg mL^{-1})	Cumulative CL2 released per 250 mg wet hydrogel (µg)
**CH–GPN–1% CL2**	0.5	-0.004	0.577	5.768
	1	0.001	0.884	9.995
	2	0.015	1.901	21.929
	3	0.018	2.113	27.858
	4	0.018	2.090	31.849
	5	0.021	2.326	38.392
**CH–GPN–2.5% CL2**	0.5	0.008	1.428	14.279
	1	0.015	1.901	21.863
	2	0.031	3.035	37.012
	3	0.038	3.532	48.047
	4	0.037	3.437	54.165
	5	0.071	5.849	85.154
**CH–GPN–5% CL2**	0.5	0.036	3.390	33.901
	1	0.066	5.518	61.957
	2	0.108	8.520	103.017
	3	0.116	9.087	125.730
	4	0.114	8.946	142.487
	5	0.189	14.241	213.333

Hydrogel formulation	Time (h)	Blank-corrected A_{280} (Abs)	Estimated CL2 concentration (µg mL^{-1})	Cumulative CL2 released per 250 mg wet hydrogel (µg)
CH–GPN–10% CL2	0.5	0.054	4.643	46.430
	1	0.131	10.104	110.326
	2	0.226	16.842	197.910
	3	0.239	17.764	240.813
	4	0.237	17.669	275.395
	5	0.220	16.440	298.440
CH–GPN–15% CL2	0.5	0.173	13.083	130.827
	1	0.269	19.915	225.314
	2	0.486	35.329	419.281
	3	0.457	33.272	469.371
	4	0.418	30.482	508.019
	5	0.358	26.227	526.430
CH–GPN–20% CL2	0.5	0.177	13.414	134.137
	1	0.485	35.210	378.931
	2	0.576	41.712	514.364
	3	0.637	46.014	640.813
	4	0.567	41.073	683.433
	5	0.517	37.527	730.118

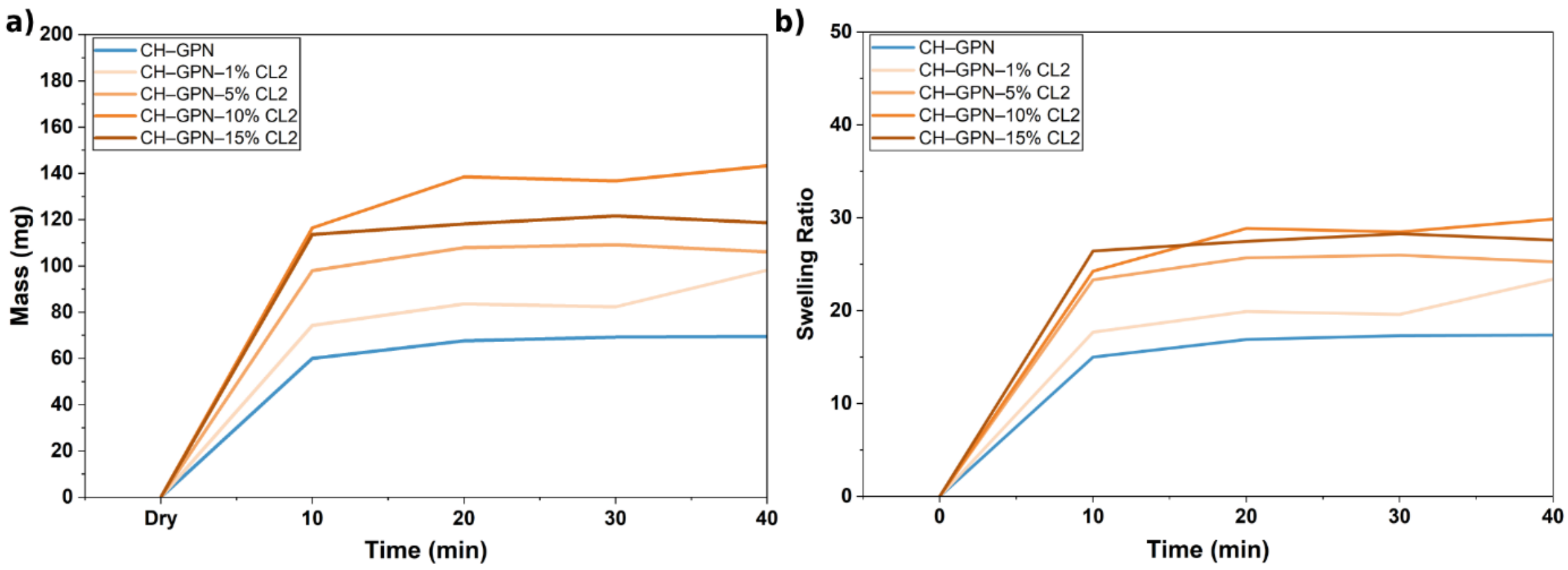

Figure S15. Water uptake and swelling behavior of lyophilized CH–GPN and CH–GPN–CL2 hydrogels. **a)** Water uptake and **b)** swelling ratio of lyophilized CH–GPN and CH–GPN–CL2 hydrogels after immersion in water for 10, 20, 30, and 40 min. Water uptake was calculated as the difference between the swollen mass and the initial dry mass. The swelling ratio was calculated as $(m_t - m_0)/m_0$, where m_0 is the dry mass and m_t is the swollen mass at each time point. Data represent one hydrogel piece per formulation.

Table S8. Raw mass, water uptake, and swelling ratio data for lyophilized CH–GPN and CH–GPN–CL2 hydrogels.

Part A. Raw mass data

Hydrogel	Mass (mg)				
	Dry	10 min	20 min	30 min	40 min
**CH–GPN**	4.0	64.0	71.6	73.2	73.5
**CH–GPN–1% CL2**	4.2	78.5	87.8	86.5	102.4
**CH–GPN–5% CL2**	4.2	102.1	112.1	113.3	110.3
**CH–GPN–10% CL2**	4.8	121.2	143.3	141.5	148.1
**CH–GPN–15% CL2**	4.3	117.9	122.4	125.9	123.0

Part B. Calculated water uptake and swelling ratio

Hydrogel	Water Uptake (mg)			
	10 min	20 min	30 min	40 min
**CH–GPN**	60.0	67.6	69.2	69.5
**CH–GPN–1% CL2**	74.3	83.6	82.3	98.2
**CH–GPN–5% CL2**	97.9	107.9	109.1	106.1
**CH–GPN–10% CL2**	116.4	138.5	136.7	143.3
**CH–GPN–15% CL2**	113.6	118.1	121.6	118.7
**Hydrogel**	**Swelling Ratio**			
	**10 min**	**20 min**	**30 min**	**40 min**
**CH–GPN**	15.0	16.9	17.3	17.4
**CH–GPN–1% CL2**	17.7	19.9	19.6	23.4
**CH–GPN–5% CL2**	23.3	25.7	26.0	25.3
**CH–GPN–10% CL2**	24.3	28.9	28.5	29.9
**CH–GPN–15% CL2**	26.4	27.5	28.3	27.6

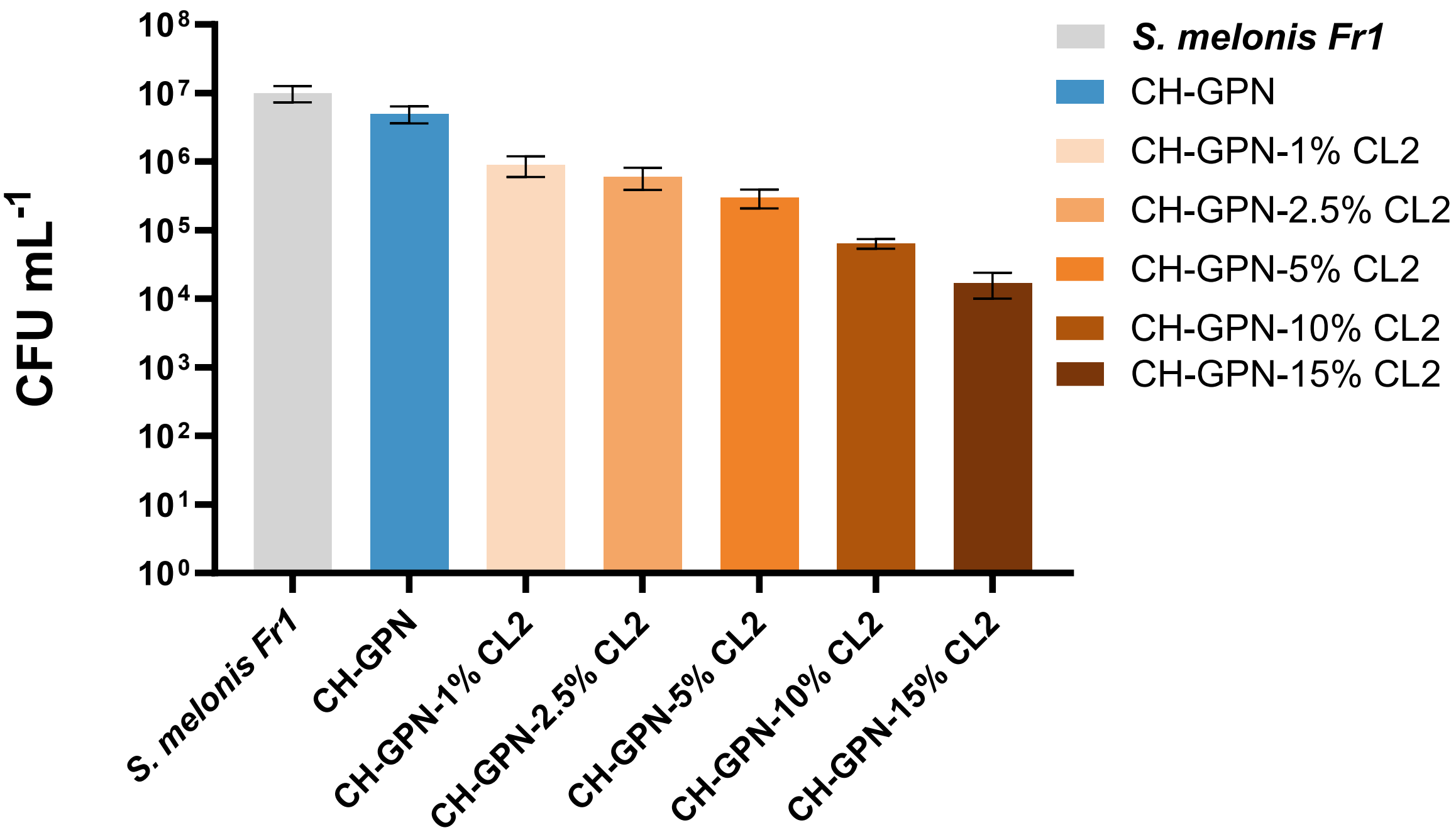

Figure S16. Bacterial inhibition of the beneficial plant-associated bacterium *Sphingomonas melonis* Fr1 by CH–GPN and CH–GPN–CL2 hydrogels containing different CL2 contents. Data are shown as CFU mL^{-1} after exposure to the hydrogel samples. Error bars represent ± SD from three independent biological replicates, each consisting of three technical replicates.

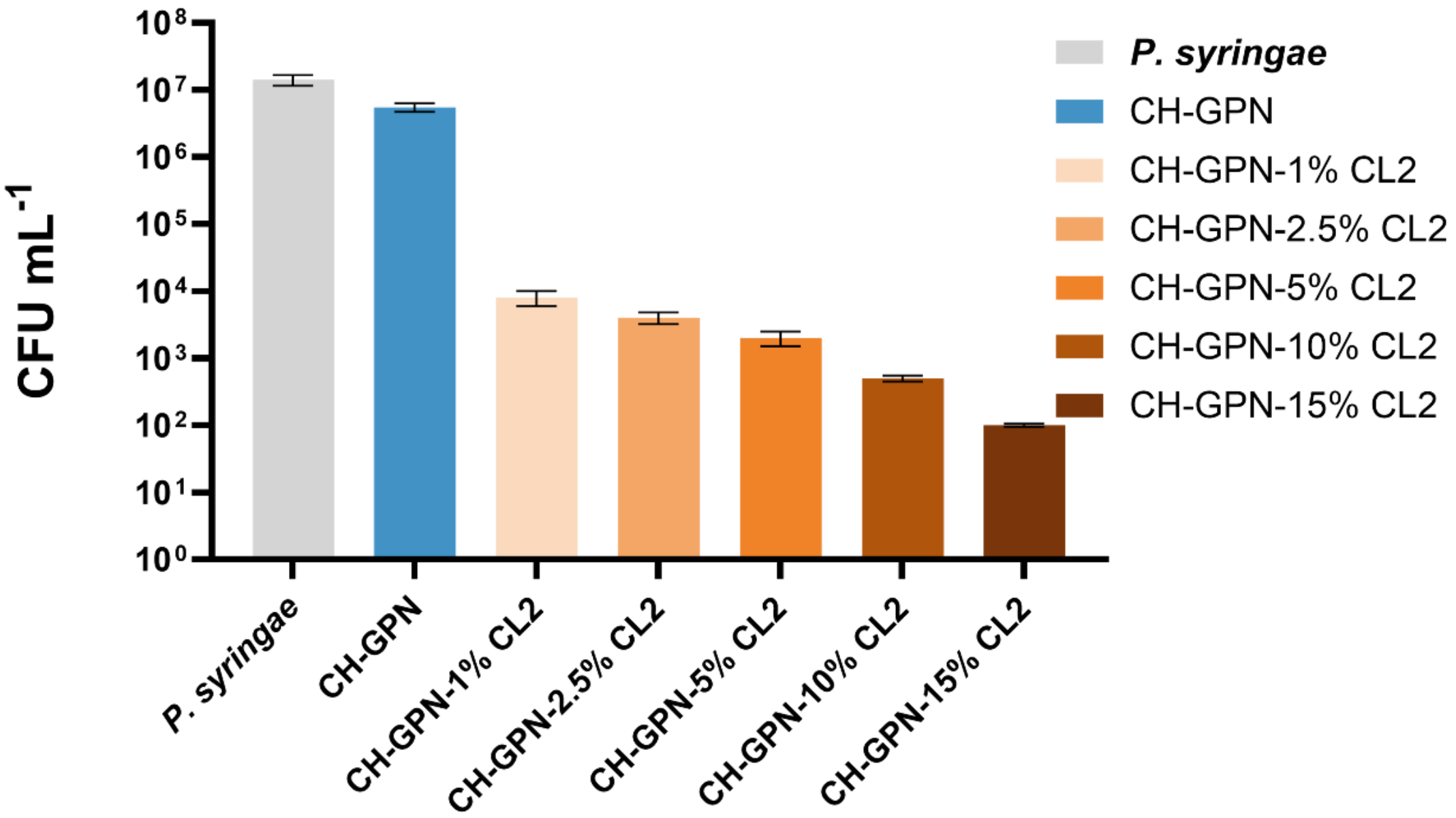

Figure S17. Antibacterial activity of CH–GPN and CH–GPN–CL2 hydrogels after two wetting/drying cycles. CH–GPN and CH–GPN–CL2 hydrogels containing different CL2 contents were subjected to two wetting/drying cycles before testing against *Pseudomonas syringae* pv. *tomato* DC3000. Data are shown as CFU mL^{-1} after exposure to the hydrogel samples. Error bars represent ± SD from three independent biological replicates, each consisting of three technical replicates.
